# Supplementary material for: Evolutionary insights into primate skeletal gene regulation using a comparative cell culture model
Source: PLoS Genet. 2022 Mar 9;18(3):e1010073. doi: 10.1371/journal.pgen.1010073 (PMC8936463; doi:10.1371/journal.pgen.1010073)
Supplement: S1 Text — (PDF) [file pgen.1010073.s001.pdf]

Supplemental Text

Supplemental Methods ... 2
Standard Validation MSC Differentiation ... 2
Standard Validation of Multipotent Cell Differentiation Potential using Histology ... 3
Cell Dissociations for Single-Cell RNA Sequencing ... 4
Assignment of 10X Single-Cell Barcodes to Species ... 4
Single-Cell Data Processing ... 5
Single-Cell Data Integration ... 5
Single-Cell Data Annotation ... 6
Topic Modeling of Single-Cell Data ... 8
Differential Expression Analyses ... 8
Gene Expression Variance ... 9
Concordance and Functional Enrichment Tests ... 9
Supplemental Results ... 10
Confirmation of MSC Differentiation and Multipotent Differentiation Potential ... 10
Single-Cell Data Balance and Reproducibility ... 10
Examination of Alternative Cell Annotations as Compared to Stages of Differentiation ... 10
DE Genes across Stages of Differentiation ... 11
DE Genes in an Alternative General Cell Classification System (Unsupervised Clustering) ... 11
DE Genes in an Alternative General Cell Classification System (Ad Hoc Assignments) ... 12
Comparison of Methods to Assign Osteogenic Cell Annotations ... 13
DE Genes across Stages of Osteogenesis ... 13
DE Genes in an Alternative Osteogenic Cell Classification System (Unsupervised Clustering) ... 14
References ... 15

All data processing and analytical scripts can be found at: https://github.com/ghousman/human-chimp-skeletal-scRNA.

Supplemental Methods

Standard Validation MSC Differentiation
The differentiation of iPSC lines into MSCs was validated using criteria outlined by the International Society for Cell Therapy (**ISCT**) [1]. Specifically, MSCs must be plastic-adherent, express a defined set of cell surface markers, and display osteogenic, adipogenic, and chondrogenic potential. These features were assessed in human and chimpanzee iPSC-derived MSCs that were used in this study, and results confirm that iPSCs were successfully differentiated into MSCs.

Morphological Changes
The morphology of iPSCs and MSCs were observed by phase contrast imaging using an EVOS M5000 Imaging System (Thermo Fisher Scientific, Waltham, MA, USA).

Cell Surface Marker Changes
Differentiation of iPSCs into MSCs was validated using flow cytometry targeting MSC markers that have been previously described by the ISCT criteria [1]. Specifically, the MSC Analysis Kit (562245, BD Biosciences, San Jose, CA, USA) was used to detect the presence or absence of CD90, CD73, CD105, CD45, CD34, CD14 or CD11b, CD19, and HLA-DR surface molecules. This kit was combined with the Zombie Violet Fixable Viability Kit (423113, BioLegend, San Diego, CA, USA) in order to also account for cell viability.

Triplicates of each iPSC and MSC line were stained according to manufacturer instructions, and flow cytometry was performed on a BD Bioscience LSRII at the Cytometry and Antibody Technology Facility (RRID: SCR_017760) at the University of Chicago. Data were analyzed using BD FACS Diva software (BD Biosciences) and FCS Express 6 (De Novo Software, Pasadena, CA, USA).

Osteogenic, Chondrogenic, and Adipogenic Potential
iPSC-derived MSCs underwent osteogenic, chondrogenic, and adipogenic differentiations using protocols modified from [2] in order to assess their potential to differentiate into each of these lineages. The osteogenic differentiation protocol is described in the manuscript, and the other protocols are described as follows.

For adipogenic differentiations, iPSC-derived MSCs were cultured to at least passage 6 and seeded at 6.8 x 10^4 cell/cm^2 in uncoated polystyrene culture dishes (4-10cm diameter). After culturing cells for 1 day in MSC medium, this medium was replaced with adipogenic medium that contained DMEM (11965-092, Thermo Fisher Scientific) supplemented with 10% FBS (FB5001, Thomas Scientific, Swedesboro, NJ, USA), 1% Penicillin/Streptomycin (30-002-CI, Corning, Bedford, MA, USA), 2mM GlutaMax (35050-061, Thermo Fisher Scientific), 10ug/mL Insulin (I2643, Sigma-Aldrich, St Louis, MO, USA), 500uM 3-Isobuthy-1-Methylxanthine (I5879, Sigma-Aldrich), 100uM Indomethacin (I8280, Sigma-Aldrich), and 100nM Dexamethasone (D4902 or D1756, Sigma-Aldrich). Cells were cultured at 37˚C in 5% CO_2 and atmospheric O_2, and medium was changed every 2-3 days for 3 weeks.

For chondrogenic differentiations, iPSC-derived MSCs were cultured to at least passage 6 and seeded at 5.0 x 10^4 cell/cm^2 in culture dishes (4-10cm diameter) that were coated in 5ug/cm^2 Type I Collagen (sc-136157, Santa Cruz Biotechnology, Dallas, TX, USA). After culturing cells for 1 day in MSC medium, this medium was replaced with chondrogenic medium that contained DMEM (11965-092, Thermo Fisher Scientific) supplemented with 50ug/mL Vitamin C (A4034 or A8960, Sigma-Aldrich), 2mM GlutaMax (35050-061, Thermo Fisher Scientific), 40ug/mL L-Proline (P5607, Sigma-Aldrich), 100ug/mL Sodium Pyruvate (11360-070, Thermo Fisher Scientific), 100nM Dexamethasone (D4902 or D1756, Sigma-Aldrich), 1X ITS Premix (354352, Corning, Bedford, MA, USA), 10ng/mL TGF-β3 (243-B3, R&D Systems, Minneapolis, MN, USA). Cells were cultured at 37˚C in 5% CO_2 and atmospheric O_2, and medium was changed every 2-3 days for 3 weeks.

Validation of osteogenic, chondrogenic, and adipogenic differentiation potential is described in the following sections.

Standard Validation of Multipotent Cell Differentiation Potential using Histology
MSCs, osteogenic cells, adipogenic cells, and chondrogenic cells were fixed using 4% paraformaldehyde in phosphate-buffered saline (sc-281692, Santa Cruz Biotechnology) and stored at 4°C until ready for staining. Once stained, cells were imaged using an Olympus SZX7 Stereo Microscope (Olympus, Center Valley, PA, USA) or an EVOS M5000 Imaging System (Thermo Fisher Scientific).

Validation of Osteogenic Differentiation using Alizarin Red Staining
Fixed osteogenic cells and MSCs were washed with 1X PBS and stained with Alizarin Red S solution (20mg/mL in DI water, pH 4.1-4.3; A5533, Sigma-Aldrich) for 45 minutes at room temperature. The stain was removed, and cells were washed with DI water to remove the excess stain prior to imaging. Alizarin Red S stains calcium deposits a red-orange color. After imaging, stain was extracted by adding a hexadecylpyridinium chloride solution and agitating for 1 hour on a shaker. The absorbance (562nm; 655090, Greiner Bio-One, Monroe, NC, USA) was measured for replicates of the extracted stain in order to quantify variation in calcium production. Statistical significance between osteogenic and MSC absorbance levels was calculated within each species and individual using one-sided Mann Whitney tests (see 10x-view-validation-data.Rmd for code).

Validation of Adipogenic Differentiation using Oil Red Staining
Fixed adipogenic cells and MSCs were washed with 1X PBS, dehydrated briefly with ethanol, and stained with Oil Red O (5mg/mL in isopropanol; 01794 Chem-Impex International Inc., Bensenville, IL, USA) for 15 minutes at room temperature. The stain was removed, and cells were briefly dehydrated with ethanol before being washed with DI water to remove the excess stains prior to imaging. Oil Red O stains intracellular lipids and fat vacuoles a red color. After imaging, stain was extracted by adding isopropanol, incubating 5 minutes at room temperature, and vortexing 15-30 seconds. The absorbance (510nm; 655090, Greiner Bio-One) was measured for replicates of the extracted stain in order to quantify variation in lipid production. Statistical significance between adipogenic and MSC absorbance levels was calculated within each species using one-sided Mann Whitney tests (see 10x-view-validation-data.Rmd for code).

Validation of Chondrogenic Differentiation using Alcian Blue Staining
Fixed chondrogenic cells and MSCs were dehydrated using a series of ethanol washes and then rehydrated with DI water before staining with Alcian Blue (5mg/mL in 0.1M HCl; A3157, Sigma-Aldrich) for 30 minutes at room temperature. The stain was removed, and cells were washed with DI water to remove the excess stains prior to imaging. Alcian Blue stains proteoglycans, which are found in connective tissue like cartilage, a blue color.

Standard Validation of Cell Differentiation Potential using qPCR
Quantitative real-time PCR (**qPCR**) was also used to confirm the osteogenic, adipogenic, and chondrogenic differentiation potential of iPSC-derived MSCs. Gene expression levels of MSCs and differentiated cells were assessed for several cell type markers. Primer sequences are provided in the following table.

Gene	Forward Primer Sequence (5' to 3')	Reverse Primer Sequence (5' to 3')
COL1A1	AGGGCCAAGACGAAGACATC	AGATCACGTCATCGCACAAC
BGLAP	CGCCTGGGTCTCTTCACTAC	CTCACACTCCTCGCCCTATT
RUNX2	CAGTAGATGGACCTCGGGAA	CCTAAATCACTGAGGCGGTC
FABP4	ACTGGGCCAGGAATTTGACG	CCCCATCTAAGGTTATGGTGCTC
PPARG	TCAGGTTTGGGCGGATGC	TCAGCGGGAAGGACTTTATGTATG
COL2A1	GGCAATAGCAGGTTCACGTACA	CGATAACAGTCTTGCCCCACTT
SOX9	GTACCCGCACTTGCACAAC	TCTCGCTCTCGTTCAGAAGTC
COL11A1	TGGTGATCAGAATCAGAAGTTCG	AGGAGAGTTGAGAATTGGGAATC
GAPDH	ATGGGGAAGGTGAAGGTCGGAGTCA	TCCTGGAAGATGGTGATGGGATTTC
GUSB	CTCATTTGGAATTTTGCCGATT	CCGAGTGAAGATCCCCTTTTTA

Briefly, total cellular RNA was extracted from samples using the ZR-Duet™ DNA/RNA MiniPrep (D7001, Zymo Research, Irvine, CA, USA) with a DNase (E1010, Zymo Research) treatment, and cDNA was prepared using the Maxima FS cDNA kit for RT-PCR (K1642, Thermo Fisher Scientific). qPCRs were run using the PowerUp™ SYBR™ Green Master Mix (A25742,

Thermo Fisher Scientific) on an Applied Biosystems QuantStudio 6 Flex Real-Time PCR System. Expression data were collected as Ct values and relative quantification levels were calculated using the $E^{-\Delta\Delta C_T}$ method [3] which determines the deviation between sample replicate Ct values and a control sample Ct value (MSCs), normalizes the target gene to a reference gene (average expression of GAPDH and GUSB), and accounts for the qPCR efficiency of each gene calculated using in-house serial dilution standards. Statistical significance between the relative quantification levels of osteogenic, adipogenic, and chondrogenic cells and those of MSCs was calculated across individuals and species using one-sided t-tests on ΔΔCt values (see 10x-view-validation-data.Rmd for code).

Cell Dissociations for Single-Cell RNA Sequencing
Cells used for single-cell RNA sequencing were first washed with 1X PBS (21-040-CV, Thermo Fisher Scientific) and then dissociated from adherent conditions into single-cell suspensions. For iPSCs, cells were incubated in a dissociation reagent (0.25mM EDTA, 150mM NaCl in PBS) for 2-5 minutes, pelleted at 1000 rpm for 5 minutes, and resuspended in a buffer solution (561527, BD Biosciences). For MSCs, cells were incubated in 0.05% Trypsin (25-052-CI, Corning) for 5-7 minutes. Once the cells detached, trypsin was neutralized with MSC medium, and the cells were pelleted at 1000 rpm for 5 minutes and resuspended in a buffer solution (561527, BD Biosciences). For osteogenic cells, cells were first incubated in 1mg/mL collagenase II (LS004204, Worthington Biochemical Corporation, Lakewood, NJ, USA) in 1X HBSS (21-022-CV, Thermo Fisher Scientific) for 10 minutes. Collagenase II was neutralized with MSC medium and removed. After a second 1X PBS wash, cells were incubated in 0.25% Trypsin (25-053-CI, Corning) for 10 minutes. Once the cells detached, trypsin was neutralized with MSC medium, and the cells were pelleted at 1000 rpm for 5 minutes and resuspended in a buffer solution (561527, BD Biosciences).

Assignment of 10X Single-Cell Barcodes to Species
Single-cell 10X cell barcodes were bioinformatically reassigned to their species of origin using standard 10X Genomics Cell Ranger 3.1.0 pipelines (Cell Ranger) [4], with the exception that reads were aligned to both the human genome (hg38) and the chimpanzee genome (panTro6) and a curated set of orthologous exons [5] was used for transcriptome alignment. Briefly, Cell Ranger aligns reads to each of two genomes, retaining only those reads that specifically align to one genome and discarding reads that align to both genomes. Each cell barcode is then classified as human or chimpanzee based on which genome has more aligned UMI counts or as a multiplet if the human UMI counts and the chimpanzee UMI counts exceed the 10th percentile of each species' distribution.

Because Cell Ranger was optimized for mixtures of human and mouse cells, which are more genetically divergent than humans and chimpanzees, we performed additional tests to determine how accurate Cell Ranger is at assigning human and chimpanzee cells and identified more accurate cell assignment criteria which we used for our data. Specifically, we ran Cell Ranger as described above on one dataset from our group that contained only human cells [YG-AH-2S-ANT-1] [6], one dataset that contained only chimpanzee cells from an external group [SRR8403265] [7], and one dataset that contained only human cells from the same external group [SRR8403264] [7]. Cell Ranger assigns most of the cell barcodes in these datasets as multiplets (**S4A, S4B, and S4C Figs**) even though the majority of UMI counts resulting from the human-only datasets were specific to the human genome and the majority of UMI counts resulting from the chimpanzee-only dataset were specific to the chimpanzee genome (**S4D, S4E, and S4F Figs**). On average, about 3% of UMIs from the human-only dataset align to the chimpanzee genome and about 4% of UMIs from the chimpanzee-only dataset align to the human genome (**S4D, S4E, and S4F Figs**). We were able to correctly assign all cells in these datasets when considering the ratio of human-aligned UMIs to the total number of aligned UMIs within each cell. Specifically, using a ratio cutoff of 0.94 successfully assigned all cells from the human-only datasets as human, and using a ratio cutoff of 0.09 successfully assigned all cells from the chimpanzee-only datasets as chimpanzees (**S4G, S4H, and S4I Figs**). These cutoffs account for the chance that human reads may align to the chimpanzee genome and that chimpanzee reads may align to the human genome due to high sequence similarity and differences in reference annotation qualities. Thus, they are the minimum criteria for assigning human and chimpanzee cells in single-cell mixtures.

However, for actual human and chimpanzee single-cell mixtures, it is also expected that there will be additional background human and chimpanzee reads due to cells that lyse before droplet formation. This background will vary from batch to batch, making it difficult to explicitly calculate. Thus, for the data collected in this study, we opted to slightly increase the ratio cutoffs to 0.9 and 0.1 for humans and chimpanzees, respectively. Using these cutoffs greatly

improves species assignments as compared to those produced directly from Cell Ranger (**S5 Fig**) (see 10x-data-0-process-test.Rmd for code).

Single-Cell Data Processing

As described above, the standard Cell Ranger pipeline using both human and chimpanzee genomes to determine species assignments discards reads that align to both genomes. Because the human and chimpanzee genomes have a high sequence similarity, using this pipeline discards an extremely high number of reads, making downstream data analyses extremely difficult.

Thus, to improve the number of reads retained for downstream analyses, we performed two additional runs of the Cell Ranger pipeline such that raw single-cell RNA sequencing reads were either aligned to the human genome (hg38) or the chimpanzee genome (panTro6) and then subsequently aligned to a curated set of orthologous exons [5] (see 10x-data-1-proces.Rmd for code). Specifically, for 10X barcodes that were assigned human as described above, reads were aligned to the human genome and orthologous exons to produce gene count matrices. Alternatively, for 10X barcodes that were assigned chimpanzee as described above, reads were aligned to the chimpanzee genome and orthologous exons to produce gene count matrices.

These matrices containing gene counts per 10X barcode were then combined and further processed using the Seurat package (v3.1.2) [8,9]. In particular, cells assigned as multiplets were removed, and cells were filtered if fewer than 1000 UMIs were detected, if fewer than 700 genes were detected, and if more than 25% of reads mapped to the mitochondria. This resulted in a total of 101,000 cells that were used in subsequent analyses (see 10x-data-1-process.Rmd and 10x-data-2-qc.Rmd for code).

Of note, we recovered substantially fewer human osteogenic cells (n=12,393) than chimpanzee osteogenic cells (n=15,791), which was likely due to cell viability differences between species following dissociations (**S7 Fig**). Specifically, the average viability of human osteogenic cells (44.86%) was lower than that of chimpanzee osteogenic cells (71.93%). Additionally, cell lines with low viability had correspondingly low cell counts (see Time 2 cells from H3: n=251, H4: n=841, H5: n=1399, and C3: n=1273 in **S7 Fig**). Viability may have been lower in human osteogenic cells because these cells progressed further through osteogenesis and produced more calcium-containing extracellular matrices, which would have made dissociating cells more difficult and more likely to result in cell death. Conversely, viability may have been higher in chimpanzee osteogenic cells because they did not progress as far through osteogenesis and had reduced calcium-containing extracellular matrices, making them easier to dissociate with a high viability.

Single-Cell Data Integration

After processing (see 10x-data-1-process.Rmd for code) and filtering (see 10x-data-2-qc.Rmd for code), data were integrated using Seurat (v3.1.2) [8,9] (see 10x-data-3-integrate.Rmd for code). This involved a series of steps, starting with data normalization. Data were log-normalized using the NormalizeData function in Seurat, which divides gene counts by the total counts, multiplies them by a scale factor of 10,000, and then natural-log transforms them.

Following this, cell cycle scores were calculated using the CellCycleScoring function in Seurat. Specifically, cell cycle scores were assigned to cells based on G2/M and S phase markers in each cell. The calculations depend on the average gene expression levels across all the cells in a dataset, so data subsetting was an important consideration. Because we anticipated that cell cycle would vary across different time points (iPSCs and MSCs continuing to replicate while late stage osteogenic cells would halt replication), cell cycle scores were calculated separately in datasets comprising cells grouped by cell line and cell type (n=42).

Finally, in order to classify cell types across collections and species, data were integrated. Integration was performed because simple data merging retained unwanted technical variation and did not fully integrate species. Even merging datasets using the intersect of the most variable genes in humans and chimpanzees did not fully integrate data across species. Thus, data were instead integrated in Seurat.

Successful data integration is dependent on how the data are subsetted and integrated across. In order to remove unwanted technical variation and allow for the successful calling of cell types using biological variation of interest, we decided to use the datasets described above (n=42) but merge those coming from the same cell line and replicate (n=14). Thus, an individual dataset comprised all cells (iPSCs, MSCs, and osteogenic cells) derived from an individual cell line replicate. Additional subsetting schemes were considered, including grouping cells by species (n=2), grouping cells by species of origin and cell type (n=6), grouping cells by 10X collection batch (n=7), grouping cells by 10X collection batch and cell type (n=21), and grouping cells by cell line and cell type (n=42). However, these alternative schemes result in cell integrations that do not fully remove batch and individual effects.

Specifically, datasets were integrated across individuals and collection batches using the reference-based, reciprocal PCA method in Seurat. This method, while perhaps less accurate than canonical correlation analysis, is computationally efficient, which was necessary for the large number of cells considered in this study. In this method, reciprocal PCA is performed to identify an effective space in which to find anchors. Specifically, each dataset is projected into every other dataset's PCA space, and anchors are constrained by the same mutual neighborhood requirement. We defined integration anchors as all genes that had non-zero UMI counts (n=18,482), as this resulted in better integration than other gene subsets tested. Because subsets of databases are assigned as references, each dataset is compared to the references instead of performing all pairwise comparisons. For these data, H1-r2 and C1-r2 were defined as references because they differentiated well based on standard validation metrics and they served as replicate samples in the study. During integration, the anchors between datasets (which represent pairwise correspondences between individual cells) were then used to harmonize the datasets (transfer information from one dataset to another).

Although a substantial amount of unwanted variation was removed from these integrated data, integrated data reductions were still correlated with UMI counts and percent of mitochondrial reads. Thus, this variation was regressed out using the ScaleData function in Seurat. Cell cycle phase was also correlated with integrated data reductions, but this variation was not regressed out because this is not recommended for differentiating cells.

Single-Cell Data Annotation
Cell type classifications were defined and assigned to single-cell data using several methods (see 10x-data-4-classification-tot.Rmd and 10x-data-plot-classification.Rmd for code). The methods reported in the manuscript include the stage of differentiation at which cells were collected, unsupervised clustering, and ad hoc assignments. Additionally, modifications to parameters in each of these classification schemes were also examined, as well as several other classification schemes.

Unsupervised Clustering
For unsupervised clustering (see 10x-data-plot-classification-clusters.Rmd for code) beyond the resolutions of 0.05, 0.25, and 0.50 that are discussed in the manuscript, resolutions of 0.10, 0.75, and 1.00 were also considered. Additionally, these higher resolutions were used to isolated osteogenic-specific clusters. In particular, a resolution of 0.50 identifies 4 osteogenic clusters, and this annotation was considered in downstream analyses.

Ad Hoc Assignments
For ad hoc assignments (see 10x-data-plot-classification-adhoc.Rmd for code), candidate gene expression patterns were used to determine general ad hoc assignments (iPSC, MSC, or osteogenic) and osteogenic ad hoc assignments (preosteoblasts, osteoblasts, embedding osteoblasts, mineralizing osteoblasts, maturing osteocytes). In both instances, a cell was defined as positively expressing a particular gene if the expression level for that gene within the cell was greater than the mean expression level of that gene across all cells. Expression levels were based on scaled integrated data that had confounding variables regressed out, as described above. Each ad hoc assignment was defined by the expression patterns of multiple genes, as described below, and for a cell to be classified to a particular ad hoc assignment, all criteria for gene expression patterns had to be met. To be thorough, additional cutoff thresholds were considered (general ad hoc assignments: mean expression level +/- 0.01, 0.05, and 0.10; osteogenic ad hoc assignments: mean expression level +/- 0.01) with more lenient thresholds resulting in more cells classified to a particular ad hoc assignment and with more conservative thresholds resulting in fewer cells classified to given ad hoc assignments.

For general ad hoc assignments, pluripotent candidate genes included *POU5F1*, *TDGF*, *SOX2*, and *EPCAM*, mesenchymal candidate genes included *THY1*, *NT5E*, *ENG*, and *CD44*, and the primary osteogenic candidate gene included *ALPL*. Pluripotent cells were defined as those expressing any of the pluripotency candidate genes (*POU5F1*, *TDGF*, *SOX2*, *EPCAM*) but no mesenchymal-specific candidate genes (*NT5E*, *ENG*, *CD44*). Mesenchymal cells were defined as those expressing any of the mesenchymal candidate genes (*THY1*, *NT5E*, *ENG*, *CD44*) but no pluripotent candidate genes (*POU5F1*, *TDGF*, *SOX2*, *EPCAM*) or osteogenic candidate genes (*ALPL*). Finally, osteogenic cells were defined as those expressing osteogenic candidate genes (*ALPL*) but no pluripotent candidate genes (*POU5F1*, *TDGF*, *SOX2*, *EPCAM*). Additional osteogenic candidate genes (*COL1A1* and *COL1A2*) were considered but not included because these genes are also expressed in mesenchymal cells which complicated disentangling these cells.

For osteogenic ad hoc assignments, cells had to first be assigned as osteogenic using the general ad hoc criteria described above. Then cells were classified into different stages of osteogenesis (preosteoblasts, osteoblasts, embedding osteoblasts, mineralizing osteoblasts, and maturing osteocytes) using candidate genes that are known to vary in expression levels across these stages of differentiation [10]. These genes include *RUNX2*, *BGLAP*, *CAPG*, *DMP1*, *PHEX*, *MEPE*, *E11*, *SOST*, and *HYOU1*. *E11* is not a part of the curated orthologous exon set used in this study, so it was not considered for osteogenic ad hoc assignments. Additionally, some of these genes did not seem appropriate for determining classifications. Thus, we examined several different combinations of genes ranging from a combination of 8 genes which was most consistent with literature [10] to an intermediary combination of 6 genes, and lastly to a combination of 4 genes which was the simplest definition considered.

First, we examined assignments using all 8 genes that overlap with our data (**S22A Fig**). Preosteoblasts were defined as cells expressing *RUNX2* but no other osteogenic genes. Osteoblasts were defined as cells expressing *BGLAP*, having variable expression of *RUNX2*, and not expressing other osteogenic genes. Embedding osteoblasts were defined as cells expressing either *CAPG*, *DMP1*, or *PHEX*, having variable expression of *RUNX2* and *BGLAP*, and not expressing other osteogenic genes. Mineralizing osteoblasts were defined as cells expressing *MEPE*, having variable expression of *RUNX2*, *BGLAP*, *CAPG*, *DMP1*, and *PHEX*, and not expressing other osteogenic genes. Maturing osteocytes were defined as cells expressing either *SOST* or *HYOU1* and having variable expression of all other osteogenic genes. All other cells were defined as unknown osteogenic.

Next, we examined assignments using only 6 of these genes (**S22C Fig**). In this set, *CAPG* was removed because it is highly expressed in all cells (pluripotent, mesenchymal, and osteogenic cells), which diminishes its usefulness as a marker for differentiating subtle cell types. Additionally, *HYOU1* was removed because it is a heat-shock protein. While this is a potentially useful marker for maturing osteocytes *in vivo* which are within a hypoxic environment of mineralized bone matrix, its expression within *in vitro* osteogenic cells may be affected by the oxygen and temperature changes that are inherent to cell culture environments. Using this combination of 6 genes, preosteoblasts were again defined as cells expressing *RUNX2* but no other osteogenic genes. Osteoblasts were again defined as cells expressing *BGLAP*, having variable expression of *RUNX2*, and not expressing other osteogenic genes. Embedding osteoblasts were defined as cells expressing either *DMP1* or *PHEX*, having variable expression of *RUNX2* and *BGLAP*, and not expressing other osteogenic genes. Mineralizing osteoblasts were defined as cells expressing *MEPE*, having variable expression of *RUNX2*, *BGLAP*, *DMP1*, and *PHEX*, and not expressing other osteogenic genes. Maturing osteocytes were defined as cells expressing *SOST* and having variable expression of all other osteogenic genes. All other cells were defined as unknown osteogenic.

Lastly, we examined assignments using only 4 of these genes (**S22E Fig**). In this set, *DMP1* and *SOST* were removed because of their substantially lower expression levels in osteogenic cells as compared to other candidate genes, and in the case of *DMP1*, its redundant nature with *PHEX*. Using this combination of 4 genes, preosteoblasts were again defined as cells expressing *RUNX2* but no other osteogenic genes. Osteoblasts were again defined as cells expressing *BGLAP*, having variable expression of *RUNX2*, and not expressing other osteogenic genes. Embedding osteoblasts were defined as cells expressing *PHEX*, having variable expression of *RUNX2* and *BGLAP*, and not expressing other osteogenic genes. Mineralizing osteoblasts were defined as cells expressing *MEPE* and having variable expression of *RUNX2*, *BGLAP*, and *PHEX*. Maturing osteocytes were defined as cells expressing *MEPE*, not expressing *RUNX2* and *BGLAP*, and having variable expression of all other osteogenic genes. All other cells were defined as unknown osteogenic.

Because transitioning between stages of osteogenesis is gradual, we felt that flexible cell type definitions which allow for variable expression patterns of stage-specific genes that are superseded in subsequent osteogenesis stages would be more biologically relevant for interpreting a differentiation trajectory and would be better able to classify cell types. Nevertheless, for each combination of genes, we also considered more strict definitions of each cell type that did not allow for variable gene expression. These definitions reduced the overall number of cell classifications as shown in the table below and were not considered further.

Gene Set Combination	Preosteoblasts	Osteoblasts	Embedding Osteoblasts	Mineralizing Osteoblasts	Maturing Osteocytes	Unknown Osteogenic
4 genes (flexible)	2004	3878	3019	4473	2443	1758
6 genes (flexible)	1509	1664	6477	6733	193	999
8 genes (flexible)	273	372	6016	4682	6030	202
4 genes (strict)	2004	1909	969	86	313	12294
6 genes (strict)	1509	1020	755	45	45	1907
8 genes (strict)	273	215	362	25	0	2499

As shown in the above table, for our flexible cell type definitions, using a combination of 8 genes resulted in more cells assigned to late stages of osteogenesis than the other gene combination sets, a combination of 6 genes resulted in more cells assigned to intermediate stages of osteogenesis than the other gene combination sets, and a combination of 4 genes resulted in more cells assigned to early stages of osteogenesis than the other gene combination sets. Our expectation was that most osteogenic cells would be in the early stages of osteogenesis, and the osteogenic ad hoc assignments determined using a combination of 4 genes most closely matched this expectation.

To formally assess which combination of osteogenic genes determined the most biologically meaningful classifications, we examined the pairwise correlations between the whole transcriptome pseudobulk of each osteogenic ad hoc classification with the assumption that correlations should be greatest between adjacent stages of osteogenesis and decrease as the separation between stages increase (e.g., correlation should be greatest between preosteoblasts and osteoblasts, reduced between preosteoblasts and embedding osteoblasts, further reduced between preosteoblasts and mineralizing osteoblasts, and the lowest between preosteoblasts and maturing osteocytes). This pattern was only observed for osteogenic ad hoc assignments determined using 4 genes (*RUNX2*, *BGLAP*, *PHEX*, *MEPE*) (**S22B, S22D, and S22F Figs**) Of note, all the pairwise correlations are generally high, because these cell types are not substantially separated across cell differentiation space. This is in contrast to pairwise correlations between the whole transcriptome pseudobulk of each stage of differentiation (**S8A Fig**), unsupervised clusters at a resolution of 0.05 (**S8B Fig**), or general ad hoc assignments (**S8C Fig**). Based on these additional analyses, the final set of candidate genes used for defining osteogenic ad hoc assignments included *RUNX2*, *BGLAP*, *PHEX*, and *MEPE*, and these data are discussed further in the manuscript.

Topic Modeling of Single-Cell Data
Topic modeling (see 10x-data-4-classification-tot.Rmd, 10x-data-plot-classification-gom.Rmd, and 10x-data-plot-classification.Rmd for code) was performed as an alternative method to examined data structure. These methods are described in the manuscript. Specifically, 3, 4, 5, 6, 7, 8, 9, 10, 15, 20, and 30 ranks were considered.

Differential Expression Analyses
Differential expression (DE) was performed using standard tests in the dream package [11] (see 10x-data-5-diffexp.Rmd and 10x-plot-diffexp.Rmd for code) and a Bayesian clustering approach implemented by Cormotif [12] (see 10x-data-5-cormotif-tot-cnts_v2.Rmd and 10x-plot-cormotif-tot-cnts_v2.Rmd for code). These methods are described in the manuscript. Briefly, for both analyses, pseudobulk expression values were calculated as described in the manuscript, and mitochondrial genes, ribosomal genes, and genes that had an average log2 transformed CPM less than or equal to zero were removed from the data (**S10 Table**). Additionally, for standard DE tests, we also randomly subsampled cell classifications to equal numbers across species before calculating pseudobulk gene expression values and performing DE tests (see 10x-data-5-diffexp.Rmd for code). This was done to ensure that the number of cells assigned to each classification did not affect broad trends that we detected in our DE results.

Genes were classified as significantly DE if they had a false discovery rate (**FDR**) < 0.01 for standard tests or if they had a posterior probability > 0.65 for Cormotif tests. These thresholds were selected after testing several different cutoffs (FDR < 0.50, 0.40, 0.30, 0.20, 0.10, 0.05, 0.01, or 0.001; posterior probability > 0.50, 0.55, 0.60, 0.65, 0.70, 0.75, 0.80, 0.85, 0.90, 0.925, 0.95, 0.975, or 0.99) to call interspecific DE genes within stages of differentiation (see 10x-plot-diffexp-thresh.Rmd and 10x-plot-cormotif-cnts-thresh_v2.Rmd for code). For each set of interspecific DE genes identified, we performed gene set enrichment tests as described below using relevant external gene sets (**S15 Table**). We expected that previously identified interspecific DE genes in iPSCs [13,14] should only be enriched among genes we identified as interspecific DE in pluripotent cells (Time 0) and that previously identified interspecific DE genes in alternative cell and tissue types (non-pluripotent, non-mesenchymal, and non-osteogenic) [13,15] should not be enriched among any interspecific DE genes identified in this study. The FDR and posterior probability thresholds that best matched these enrichment expectations (**S14 and S17 Figs**) were then used for downstream analyses (FDR < 0.01; posterior probability > 0.65).

Gene Expression Variance

We examined whether variance in gene expression differs across cell classifications (see 10x-data-6-variance-tot.Rmd for code) as described in the manuscript.

Concordance and Functional Enrichment Tests

We assessed the enrichment of Gene Ontology categories among various sets of genes identified in this study using the enrichGO function in the clusterProfiler v3.12.0 package in R [16] (see 10x-plot-classification.Rmd and 10x-plot-enrichment-gom.Rmd for code) and the enrichment of relevant gene sets (**S15 Table**) in the DE genes in this study using a Fisher's exact test (see 10x-plot-diffexp.Rmd and 10x-plot-cormotif- cnts_v2.Rmd for code) as described in the manuscript. Because very few skeletal-trait-associated genes are known for hip geometry [17] and bone area [18], we also examined which of these skeletal-phenotype genes overlap our interspecific DE genes. In particular, out of the 10 genes associated with GWAS hits for hip geometry [17], only seven passed filtering criteria and were tested in our data (*FGFR4, NSD1, LRP5, PPP6R3, GAL, CCDC91, RUNX1*), with *FGFR4* only meeting these criteria for Cormotif DE tests within stages of differentiation. Additionally, out of the 15 genes associated with GWAS hits for bone area [18], only eight passed filtering criteria and were tested in our data (*BCKDHB, COL11A1, CTDSP2, DYM, HHIP, SH3GL3, SOX9, WNT4*), with only *BCKDHB, COL11A1, CTDSP2,* and *DYM* meeting these criteria for all of DE tests. Specifically, *HHIP* was not examined in any standard DE tests, *SH3GL3* was not examined in any standard DE tests or Cormotif DE tests within stages of osteogenesis, *SOX9* was not examined in standard DE tests within stages of osteogenesis, and *WNT4* was not examined in standard or Cormotif DE tests within stages of osteogenesis.

Supplemental Results

Confirmation of MSC Differentiation and Multipotent Differentiation Potential
Morphological and cell surface marker changes were assessed to confirm the successful differentiation of iPSCs into MSCs. Consistent with criteria outlined by the ISCT [1], all MSC lines were plastic adherent and showed characteristic elongated shapes, as opposed to the more rounded shape of iPSCs (**S1A Fig**). Additionally, iPSCs expressed CD90, MSCs expressed CD90, CD73, and CD105, and neither expressed CD45, CD34, CD14 or CD11b, CD19 and HLA-DR (**S1B and S1C Figs and S5 Table**). Altogether, these results confirm the successful differentiation of MSCs.

Osteogenic, adipogenic, and chondrogenic differentiations were performed to confirm the multipotent differentiation potential of MSCs. Validation of osteogenic differentiation via Alizarin Red staining of calcium deposits is discussed in the manuscript. In general, calcium production was greater in osteogenic cells than in MSCs across species and individuals (**Figs 1D, S1D, and S2 and S6 Table**). Additionally, adipogenic differentiation was validated using Oil Red staining which confirmed the production of fat globules, and chondrogenic differentiation was validated using Alcian Blue staining which confirmed the production of collagen extracellular matrix. In general, lipid production was greater in adipogenic cells than in MSCs across species and individuals, and proteoglycan production was greater in chondrogenic cells than in MSCs (**S1D Fig and S7 Table**). As a secondary confirmation, qPCR was used to confirm the increased expression of *COL1A1, BGLAP*, and *RUNX2* in osteogenic cells, *FABP4* and *PPARG* in adipogenic cells, and *COL2A1*, *SOX9*, and *COL11A1* in chondrogenic cells (**S1E Fig and S8 Table**).

Single-Cell Data Balance and Reproducibility
Species are fairly well integrated within each stage of differentiation (**S6A Fig**). There is some separation, but other known batch effects (**S2 and S9 Tables**) do not seem to account for these interspecific cell distribution separations. Using the gene expression patterns recovered from different technical replicates, we also found that these scRNA-seq data are highly reproducible. Specifically, we observed strong correlations between replicates when considering gene expression within each stage of differentiation, both when species are combined (**Figs 2E, 2F, and 2G**) or analyzed separately (**S6B Fig**). For these correlation tests, log-normalized scRNA-seq gene counts as described above were first averaged across cells in each technical replicate and then log-transformed. Lastly, Pearson correlations were calculated between the log-transformed average gene counts of one replicate and the log-transformed average gene counts of the second replicate for a given cell classification. Of note, the gene expression correlation of the osteogenic technical replicates is substantially lower for chimpanzees than for humans. This may be due to increased differences in cell composition and cell viability which are observed between chimpanzee replicates as compared to human replicates.

Examination of Alternative Cell Annotations as Compared to Stages of Differentiation
We used alternative classification schemes, including unsupervised clustering and ad hoc assignment methods, to annotate cells and compared these alternative annotation results to the stages of differentiation results discussed in the manuscript. Specifically, we examined whether scRNA-seq data remained balanced across species and reproducible, displayed divergent transcriptomic patterns, and exhibited expected marker gene expression levels.

Unsupervised clustering with a resolution of 0.05 identified 6 clusters – 3 pluripotent clusters (iPSC.c1, iPSC.c2, iPSC.c3), 1 mesenchymal cluster (MSC.c1), and 2 osteogenic clusters (Osteogenic.c1, Osteogenic.c2) (**S9A Fig**). Within each cluster, similar numbers of cells were recovered in each species (**S9B Fig**), and UMI counts per cell (**S9C Fig**) and gene counts per cell (**S9D Fig**) are also similar across species. Additionally, gene expression patterns recovered from different technical replicates are strongly correlated within each cluster (**S9E Fig**). Whole transcriptome pseudobulk correlations also show clear transcriptomic differences across cell classification (**S8B Fig**). Finally, cluster-specific marker genes are characterized by biologically relevant functional enrichments (**S9F, S9G, and S9H Figs**).

General ad hoc assignments classified cells into iPSCs, MSCs, and osteogenic cells as described above (**S10A Fig**). Within each cell classification, similar numbers of cells were recovered in each species (**S10B Fig**), and UMI counts per cell (**S10C Fig**) and gene counts per cell (**S10D Fig**) are also similar across species. Additionally, gene expression patterns recovered from different technical replicates are strongly correlated within each cell assignment (**S10E Fig**). Whole transcriptome

pseudobulk correlations also show clear transcriptomic differences across cell classification (**S8C Fig**). Finally, assignment-specific marker genes are characterized by biologically relevant functional enrichments (**S10F, S10G, and S10H Figs**).

Overall, the cell groupings formed when using unsupervised clustering and ad hoc assignment methods display similar characteristics to the of cell groupings based on stages of differentiation. These similarities further confirm that stages of differentiation serve as good proxies for general cell types, so this classification scheme is used for all downstream analyses described in the manuscript.

DE Genes across Stages of Differentiation

As discussed in the manuscript, interspecific DE genes were identified across stages of differentiation. Functional enrichments of these interspecific DE genes were examined in order to assess concordance with previously identified genes and to ascertain biological functions (**Figs 3E, 3F, S23A**, **S25A, and S26A and S16-S18 Tables**). Resulting trends for standard DE tests are supplied here, while details for the Cormotif DE tests are discussed in the manuscript.

As expected, genes previously identified in iPSCs as DE between species [13,14] are enriched among genes we identified as interspecific DE in pluripotent cells but not among genes classified as interspecific DE in mesenchymal or osteogenic cells. Additionally, genes previously identified as DE between species in non-pluripotent, non-mesenchymal, and non-osteogenic cell types and tissues [13,15] are not enriched among genes we identified as DE between species, with two exceptions – (1) genes previously identified as DE between species in kidney tissue [15] are enriched among genes we identified as interspecific DE in pluripotent cells and (2) genes previously identified as DE between species in liver tissue [15] are enriched among genes we identified as interspecific DE in pluripotent and mesenchymal cells. Lastly, genes previously identified as DE between species in CNCCs [14] are enriched among genes we identified as interspecific DE in mesenchymal and osteogenic cells, and interspecific differentially methylated regions (**DMRs**) in bone tissues [19] are enriched among genes we identified as interspecific DE in mesenchymal cells.

We did not find enrichment of interspecific DE genes among skeletally-relevant GO categories. However, we did find enrichment of interspecific DE genes among other GO categories, including several functions related to transcriptional regulation (**S16-S18 Tables**). Similarly, we found no overlap of skeletal phenotype-related genes among interspecific DE genes. Specifically, we found no significant enrichment of hip geometry [17], bone area [18], height [20], osteoporosis [21], or osteoarthritis [22] related loci among interspecific DE genes identified in this study. Because there are very few genes associated with GWAS hits for hip geometry [17] and bone area [18] that also overlap with genes tested in this study, we also examined overlapping genes individually. Of the hip geometry genes [17] that were tested in our data, we found that *PPP6R3* is also a interspecific DE gene that is shared between pluripotent cells and mesenchymal cells. Additionally, of the bone area genes [18] that were tested in our data, *BCKDHB* and *COL11A1* are interspecific DE genes unique to pluripotent cells.

DE Genes in an Alternative General Cell Classification System (Unsupervised Clustering)

We examined DE between species when cells were annotated using the general unsupervised clustering method described above and found that these results were similar to the stages of differentiation results discussed in the manuscript.

Specifically, within each general unsupervised cluster, we discovered hundreds of DE genes between species. Standard DE analyses of pseudobulk data detected 2,155 interspecific DE genes in iPSC.c1, 413 in iPSC.c2, 0 in iPSC.c3, 936 in MSC.c1, 534 in Osteogenic.c1, and 209 in Osteogenic.c2 with an FDR < 0.01 (**S12A Fig and S11 Table**). To complement the stages of differentiation cross-cell type comparisons discussed in the manuscript, we chose 1 pluripotent, 1 mesenchymal, and 1 osteogenic cluster to consider further. iPSC.c1 was chosen as it displayed the best pluripotent marker gene expression patters of all pluripotent clusters identified (iPSC.c1, iPSC.c2, iPSC.c3) (**S11A and S11B Figs**); MSC.c1 was chosen as it was the only mesenchymal cluster identified; and Osteogenic.c1 was chosen as it displayed the best osteogenic marker gene expression patterns of all osteogenic clusters identified (Osteogenic.c1, Osteogenic.c2) (**S11C and S11D Figs**). Focusing in on the primary cluster for each cell type (iPSC.c1, MSC.c1, Osteogenic.c1), some of these DE genes are shared across clusters, while some are specific to particular clusters (**S15B Fig**). Overall, there is a

general decrease in interspecific DE as differentiation progresses. We found similar interspecific DE results when using Cormotif [12]. In particular, we found that 5 correlation motifs best fit our pseudobulk data (**S12B Fig**). We detected 5,168 interspecific DE genes in iPSC.c1, 2,771 in iPSC.c2, 1,295 in iPSC.c1, 4,535 in MSC.c1, 3,585 in Osteogenic.c1, and 3,507 in Osteogenic.c2 with a posterior probability > 0.65 (**S16C Fig and S12 Table**), and several these DE genes are shared across the primary clusters for each cell type (**S16D Fig**).

Functional enrichments of these interspecific DE genes (standard and Cormotif) were examined in order to assess concordance with previously identified genes and to ascertain biological functions (**S12D, S12E, S23B, S25B, and S26B Figs and S16-S18 Tables**). As expected, genes previously identified in iPSCs as DE between species [13,14] are enriched among genes we identified as interspecific DE in iPSC.c1 (standard and Cormotif) but not among the interspecific DE genes we identified in other clusters (with several exceptions in the Cormotif tests). Additionally, by and large, genes previously identified as DE between species in non-pluripotent, non-mesenchymal, and non-osteogenic cell types and tissues [13,15] are not enriched among genes we identified as DE between species (with some exceptions in the standard tests and Cormotif tests). On the other hand, genes previously identified as DE between species in CNCCs [14] are enriched among genes we identified as interspecific DE in MSC.c1 and Osteogenic.c1 (standard and Cormotif), and interspecific DMRs in bone tissues [19] are enriched among genes we identified as interspecific DE in MSC.c1 (standard). Regarding skeletal phenotype-related genes, almost no enrichments are present, except for height-related loci [20] which are enriched among genes we identified as interspecific DE in iPSC.c1 and MSC.c1 (Cormotif).

Lastly, we used Cormotif [12] to test for DE genes across clusters and found that 4 correlation motifs best fit our pseudobulk data when performing pairwise tests between subsequent clusters separately for each species (**S12C Fig**). These motifs are highly conserved across species, with about 85% of DE genes between clusters conserved across species (**S13 Table**). Of the remaining DE genes, 22 DE genes detected between iPSC.c1 and MSC.c1 differ between species, and 265 DE genes detected between MSC.c1 and Osteogenic.c1 differ between species.

DE Genes in an Alternative General Cell Classification System (Ad Hoc Assignments)

We examined DE between species when cells were annotated using the general ad hoc assignment method described above and found that these results were similar to the stages of differentiation results discussed in the manuscript.

Specifically, within each general ad hoc classification, we discovered hundreds of DE genes between species. Standard DE analyses of pseudobulk data detected 2,128 interspecific DE genes in iPSCs, 963 in MSCs, and 613 in osteogenic cells with an FDR < 0.01 (**S13A Fig and S11 Table**). Some of these DE genes are shared across ad hoc assignments, while some are specific to particular assignment (**S15C Fig**). Overall, there is a general decrease in interspecific DE as differentiation progresses. We found similar interspecific DE results when using Cormotif [12]. In particular, we found that 2 correlation motifs best fit our pseudobulk data (**S13B Fig**). We detected 6,629 interspecific DE genes in iPSCs, 4,567 in MSCs, and 4,553 in osteogenic cells with a posterior probability > 0.65 (**S16E Fig and S12 Table**), and several these DE genes are shared across ad hoc assignments (**S16F Fig**).

Functional enrichments of these interspecific DE genes (standard and Cormotif) were examined in order to assess concordance with previously identified genes and to ascertain biological functions (**S13D, S13E, S23C, S25C, and S26C Figs and S16-S18 Tables**). As expected, genes previously identified in iPSCs as DE between species [13,14] were enriched among genes we identified as interspecific DE in iPSCs (standard and Cormotif) but not among the interspecific DE genes we identified in other ad hoc assignments (with some exceptions in the Cormotif tests). Additionally, by and large, genes previously identified as DE between species in non-pluripotent, non-mesenchymal, and non-osteogenic cell types and tissues [13,15] are not enriched among genes we identified as DE between species (with some exceptions in the standard tests and in Cormotif tests). On the other hand, genes previously identified as DE between species in CNCCs [14] and interspecific DMRs in bone tissues [19] are enriched among genes we identified as interspecific DE in MSCs and Osteogenic cells (standard and Cormotif). Regarding skeletal phenotype-related genes, almost no enrichments are present, except for height-related loci [20] which are enriched among genes we identified as interspecific DE in iPSCs (Cormotif).

Lastly, we used Cormotif [12] to test for DE genes across ad hoc assignments and found that 2 correlation motifs best fit our pseudobulk data when performing pairwise tests between subsequent ad hoc assignments separately for each species (**S13C Fig**). These motifs are not well conserved across species, with only 29% of DE genes between ad hoc assignments conserved across species (**S13 Table**). Of the remaining DE genes, 453 DE genes detected between iPSCs and MSCs differ between species, and 262 DE genes detected between MSCs and osteogenic cells differ between species.

Comparison of Methods to Assign Osteogenic Cell Annotations
In addition to examining DE across general stages of differentiation, we wanted to use our comparative skeletal cell culture model to more specifically study gene regulation within skeletal cell types. Grouping all osteogenic cells together in bulk provided some interspecific insights, but the increased cell heterogeneity at this time point, as evidenced by increased gene expression variance (**Figs 3D and S18**), presented a barrier. To disentangle the approximate location of cells along the course of osteogenesis, we tested several cell classification methods as described above.

Using a flexible ad hoc assignment method that considers 4 candidate genes [10], we detected 2,004 preosteoblasts, 3,878 osteoblasts, 3,019 embedding osteoblasts, 4,473 mineralizing osteoblasts, and 2,443 maturing osteocytes (**S19A Fig**). Fewer human cells are assigned to each of these stages than chimpanzee cells (**S19B Fig**), but this is primarily because fewer human cells were recovered from the osteogenic stage of differentiation collection (**Fig 2B**). Interestingly, although humans and chimpanzees have similar numbers of preosteoblasts, the distribution of cell counts along other stages of osteogenesis differs between species, with chimpanzees having an accumulation of cells at intermediate stages of osteogenesis (osteoblasts, embedding osteoblasts, and mineralizing osteoblasts) and humans having a decreased number of intermediate stages (embedding osteoblasts) but an increase in late stage cells (mineralizing osteoblasts and maturing osteocytes) (**S19B Fig**). These cell count distributions reflect the same pattern that we observed in the more traditional validation results, with chimpanzees on average producing less calcium deposits than humans (**S2 Fig**), indicating a larger proportion of osteogenic precursor cells in chimpanzees than in humans. Moreover, it suggests that perhaps human cells can transition more quickly between intermediate and later stage osteogenic cells than chimpanzee cells. Finally, because these cells were assigned to a specific stage of osteogenesis based on their expression of known marker genes (**Figs 4B and S22E**), they retain these characteristic cell-specific expression patterns (**S19C Fig**) which allow for clear biological interpretations.

Alternatively, using an unsupervised clustering method with a resolution of 0.50, we identified 4 osteogenic clusters (Osteogenic.c1, Osteogenic.c2, Osteogenic.c3, Osteogenic.c4) (**S19D Fig**). Similar to the osteogenic ad hoc assignments, fewer human cells are assigned to each osteogenic cluster than chimpanzee cells (**S19E Fig**). Again, this is because fewer human cells were recovered from the osteogenic stage of differentiation collection (**Fig 2B**). In contrast to the osteogenic ad hoc assignments, both species have similar proportions of cells assigned to each osteogenic cluster (**S19E Fig**). Although cells within these clusters display distinct patterns of candidate maker gene expression patterns (**S19F Fig**), these patterns are not always identical across species, nor do they clarify the stage at which cells are along the course of osteogenesis. Thus, because assigning cells using an ad hoc method produced more biologically meaningful groups of cells that are more consist with standard validation methods than assigning cells using clustering methods, we proceeded with this osteogenic ad hoc assignment scheme in the manuscript.

DE Genes across Stages of Osteogenesis
As discussed in the manuscript, interspecific DE genes were identified across stages of osteogenesis. Functional enrichments of these interspecific DE genes were examined in order to assess concordance with previously identified genes and to ascertain biological functions (**Figs 4E, 4F, S23D**, **S25D, and S26D and S16-S17 Tables**). Resulting trends for the standard DE tests are supplied here, while details for the Cormotif DE tests are discussed in the manuscript.

As expected, genes previously identified in iPSCs as DE between species [13,14] and genes previously identified as DE between species in non-pluripotent, non-mesenchymal, and non-osteogenic cell types and tissues [13,15] are not enriched among genes we identified as DE between species. One exception was that genes previously identified as DE between species in kidney tissue [15] are enriched among genes we identified as interspecific DE in osteoblasts and embedding osteoblasts. Conversely, although interspecific DMRs in bone tissues [19] are not enriched among genes we

identified as DE between species, genes previously identified as DE between species in CNCCs [14] are enriched among genes we identified as interspecific DE in preosteoblasts and in osteoblasts.

We found no overlap of skeletal phenotype-related genes among interspecific DE genes. Specifically, we found no significant enrichment of hip geometry [17], bone area [18], height [20], osteoporosis [21], or osteoarthritis [22] related loci among interspecific DE genes identified in this study. Because there are very few genes associated with GWAS hits for hip geometry [17] and bone area [18] that also overlap with genes tested in this study, we also examined overlapping genes individually. However, we did not identify any genes that overlap. Finally, we did not find skeletally-relevant GO category enrichment for most of our interspecific DE genes, with the exception of interspecific DE genes unique to preosteoblasts which are enriched in functions essential for skeletal tissue development and maintenance [23], such as Wnt signaling and tissue morphogenesis.

DE Genes in an Alternative Osteogenic Cell Classification System (Unsupervised Clustering)
We examined DE between species when cells were annotated using the osteogenic unsupervised clustering method described above and found that these results differed from the stages of osteogenesis results discussed in the manuscript.

Specifically, within each unsupervised osteogenic cluster, we discovered hundreds of DE genes between species. Standard DE analyses of pseudobulk data identified 643 interspecific DE genes in Osteogenic.c1, 1 in Osteogenic.c2, 428 in Osteogenic.c3, and 217 in Osteogenic.c4 with an FDR < 0.01 (**S20B Fig and S11 Table**). Some of these DE genes are shared across osteogenic clusters, while some are specific to particular clusters (**S15D Fig**). We found similar interspecific DE results when using Cormotif [12], although this method suggests a greater amount of sharing between clusters. In particular, we found that 2 correlation motifs best fit our pseudobulk data (**S20C Fig**). We identified 3,847 interspecific DE genes in Osteogenic.c1, 91 in Osteogenic.c2, 3,846 in Osteogenic.c3, and 3,813 in Osteogenic.c4 with a posterior probability > 0.65 (**S16I Fig and S12 Table**), and almost all of these DE genes are shared across clusters (**S16J Fig**).

Functional enrichments of these interspecific DE genes (standard and Cormotif) were examined in order to assess concordance with previously identified genes and to ascertain biological functions (**S20D, S20E, S23E, S25E, and S26E Figs and S16-S17 Tables**). As expected, genes previously identified in iPSCs as DE between species [13,14] and genes previously identified as DE between species in non-pluripotent, non-mesenchymal, and non-osteogenic cell types and tissues [13,15] are not enriched among genes we identified as DE between species. Conversely, although genes previously identified as DE between species in CNCCs [14] are not enriched among genes we identified as DE between species, interspecific DMRs in bone tissues [19] are enriched among genes we identified as interspecific DE in Osteogenic.c1, Osteogenic.c3, and Osteogenic.c4 (Cormotif). Nevertheless, despite these enrichments, there are no significant overlaps between our interspecific DE genes and skeletal phenotype-related genes which complicates functional interpretations.

References

1. Dominici M, Le Blanc K, Mueller I, Slaper-Cortenbach I, Marini F, Krause D, et al. Minimal criteria for defining multipotent mesenchymal stromal cells. The International Society for Cellular Therapy position statement. Cytotherapy. 2006;8: 315–317. doi:10.1080/14653240600855905

2. Nejadnik H, Diecke S, Lenkov OD, Chapelin F, Donig J, Tong X, et al. Improved Approach for Chondrogenic Differentiation of Human Induced Pluripotent Stem Cells. Stem Cell Rev. 2015;11: 242–253. doi:10.1007/s12015-014-9581-5

3. Pfaffl MW. A new mathematical model for relative quantification in real-time RT–PCR. Nucleic Acids Res. 2001;29: e45.

4. Zheng GXY, Terry JM, Belgrader P, Ryvkin P, Bent ZW, Wilson R, et al. Massively parallel digital transcriptional profiling of single cells. Nat Commun. 2017;8: 14049. doi:10.1038/ncomms14049

5. Mittleman BE, Pott S, Warland S, Barr K, Cuevas C, Gilad Y. Divergence in alternative polyadenylation contributes to gene regulatory differences between humans and chimpanzees. Coop G, Barkai N, editors. eLife. 2021;10: e62548. doi:10.7554/eLife.62548

6. Hung A, Housman G, Briscoe E, Cuevas C, Gilad Y. Characterizing gene expression responses to biomechanical strain in an in vitro model of osteoarthritis. bioRxiv. 2021; 2021.02.22.432314. doi:10.1101/2021.02.22.432314

7. Marchetto MC, Hrvoj-Mihic B, Kerman BE, Yu DX, Vadodaria KC, Linker SB, et al. Species-specific maturation profiles of human, chimpanzee and bonobo neural cells. Elife. 2019;8: e37527. doi:10.7554/eLife.37527

8. Butler A, Hoffman P, Smibert P, Papalexi E, Satija R. Integrating single-cell transcriptomic data across different conditions, technologies, and species. Nature Biotechnology. 2018 [cited 27 Apr 2018]. doi:10.1038/nbt.4096

9. Stuart T, Butler A, Hoffman P, Hafemeister C, Papalexi E, Mauck WM, et al. Comprehensive Integration of Single-Cell Data. Cell. 2019;177: 1888-1902.e21. doi:10.1016/j.cell.2019.05.031

10. Dallas SL, Prideaux M, Bonewald LF. The Osteocyte: An Endocrine Cell … and More. Endocr Rev. 2013;34: 658–690. doi:10.1210/er.2012-1026

11. Hoffman GE, Roussos P. Dream: powerful differential expression analysis for repeated measures designs. Gorodkin J, editor. Bioinformatics. 2021;37: 192–201. doi:10.1093/bioinformatics/btaa687

12. Wei Y, Tenzen T, Ji H. Joint analysis of differential gene expression in multiple studies using correlation motifs. Biostatistics. 2015;16: 31–46. doi:10.1093/biostatistics/kxu038

13. Blake LE, Thomas SM, Blischak JD, Hsiao CJ, Chavarria C, Myrthil M, et al. A comparative study of endoderm differentiation in humans and chimpanzees. Genome Biology. 2018;19: 162. doi:10.1186/s13059-018-1490-5

14. Gokhman D, Agoglia RM, Kinnebrew M, Gordon W, Sun D, Bajpai VK, et al. Human–chimpanzee fused cells reveal cis -regulatory divergence underlying skeletal evolution. Nature Genetics. 2021; 1–10. doi:10.1038/s41588-021-00804-3

15. Blake LE, Roux J, Hernando-Herraez I, Banovich NE, Perez RG, Hsiao CJ, et al. A comparison of gene expression and DNA methylation patterns across tissues and species. Genome Res. 2020;30: 250–262. doi:10.1101/gr.254904.119

16. Yu G, Wang L-G, Han Y, He Q-Y. clusterProfiler: an R Package for Comparing Biological Themes Among Gene Clusters. OMICS. 2012;16: 284–287. doi:10.1089/omi.2011.0118

17. Hsu Y-H, Estrada K, Evangelou E, Ackert-Bicknell C, Akesson K, Beck T, et al. Meta-Analysis of Genomewide Association Studies Reveals Genetic Variants for Hip Bone Geometry. Journal of Bone and Mineral Research. 2019;34: e3698. doi:10.1002/jbmr.3698

18. Styrkarsdottir U, Stefansson OA, Gunnarsdottir K, Thorleifsson G, Lund SH, Stefansdottir L, et al. GWAS of bone size yields twelve loci that also affect height, BMD, osteoarthritis or fractures. Nature Communications. 2019;10: 2054. doi:10.1038/s41467-019-09860-0

19. Gokhman D, Nissim-Rafinia M, Agranat-Tamir L, Housman G, García-Pérez R, Lizano E, et al. Differential DNA methylation of vocal and facial anatomy genes in modern humans. Nature Communications. 2020;11: 1–21. doi:10.1038/s41467-020-15020-6

20. Yengo L, Sidorenko J, Kemper KE, Zheng Z, Wood AR, Weedon MN, et al. Meta-analysis of genome-wide association studies for height and body mass index in ∼700000 individuals of European ancestry. Hum Mol Genet. 2018;27: 3641–3649. doi:10.1093/hmg/ddy271

21. Morris JA, Kemp JP, Youlten SE, Laurent L, Logan JG, Chai RC, et al. An atlas of genetic influences on osteoporosis in humans and mice. Nat Genet. 2019;51: 258–266. doi:10.1038/s41588-018-0302-x

22. Steinberg J, Southam L, Butterfield NC, Roumeliotis TI, Fontalis A, Clark MJ, et al. Decoding the genomic basis of osteoarthritis. bioRxiv. 2020; 835850. doi:10.1101/835850

23. Baron R, Kneissel M. WNT signaling in bone homeostasis and disease: from human mutations to treatments. Nat Med. 2013;19: 179–192. doi:10.1038/nm.3074
