# Supplementary figures and images for: Evolutionary insights into primate skeletal gene regulation using a comparative cell culture model
Source: PLoS Genet. 2022 Mar 9;18(3):e1010073. doi: 10.1371/journal.pgen.1010073 (PMC8936463; doi:10.1371/journal.pgen.1010073)

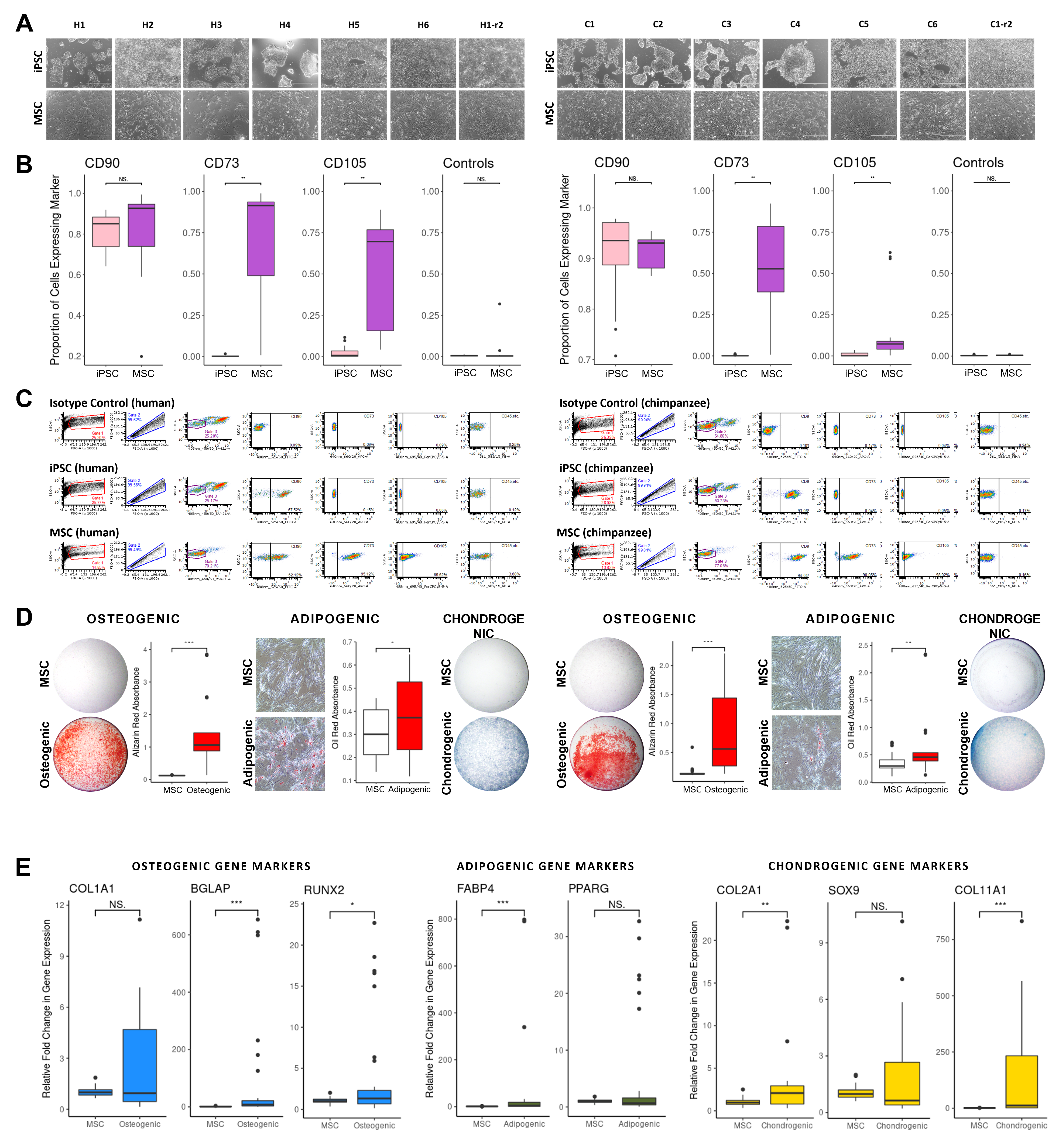

Supplement: S1 Fig — (A) Phase contrast imaging at 4X of each cell line when pluripotent (Time 0, top row) and mesenchymal (Time 1, bottom row). (B) Box plots showing the proportion of cells expressing CD90, CD73, CD105, or control markers (CD45, CD34, CD14 or CD11b, CD19, and HLA-DR) for pluripotent cells (Time 0, pink) and mesenchymal cells (Time 1, purple) from all human biological and technical replicates (left set of plots) and all chimpanzee biological and technical replicates (right set of plots). Statistical significance was determined using one-sided Mann Whitney tests. (C) Representative flow cytometry analysis in one human cell line (left) and one chimpanzee cell line (right). Each set of plots displays results for the isotype control (top row), pluripotent cells (Time 0, second row), and mesenchymal cells (Time 1, bottom row). Plots in each row from left to right: (1) forward scatter area versus side scatter area of total cells, (2) forward scatter area versus forward scatter height of Gate 1 cells, (3) Zombie Violet fluorescence versus side scatter area of Gate 2 cells, (4) CD90 FITC fluorescence versus side scatter area of Gate 3 cells, (5) CD73 APC fluorescence versus side scatter area of Gate 3 cells, (6) CD105 PerCPCy5.5 fluorescence versus side scatter area of Gate 3 cells, (7) control marker (CD45, CD34, CD14 or CD11b, CD19, and HLA-DR) PE fluorescence versus side scatter area of Gate 3 cells. (D) Histological validation of MSC differentiation potential in human cell lines (left set of plots) and chimpanzee cell lines (right set of plots). Plots in each set from left to right: (1) images of mesenchymal cells (Time 1) and osteogenic cells (Time 2) after Alizarin Red staining (image zoomed out to display the entire cell culture well), (2) box plot of absorbance values of extracted Alizarin Red stain from all biological and technical replicates from a given species, (3) phase contrast image at 4X magnification of mesenchymal cells (Time 1) and adipogenic cells after Oil [file pgen.1010073.s020.tif]

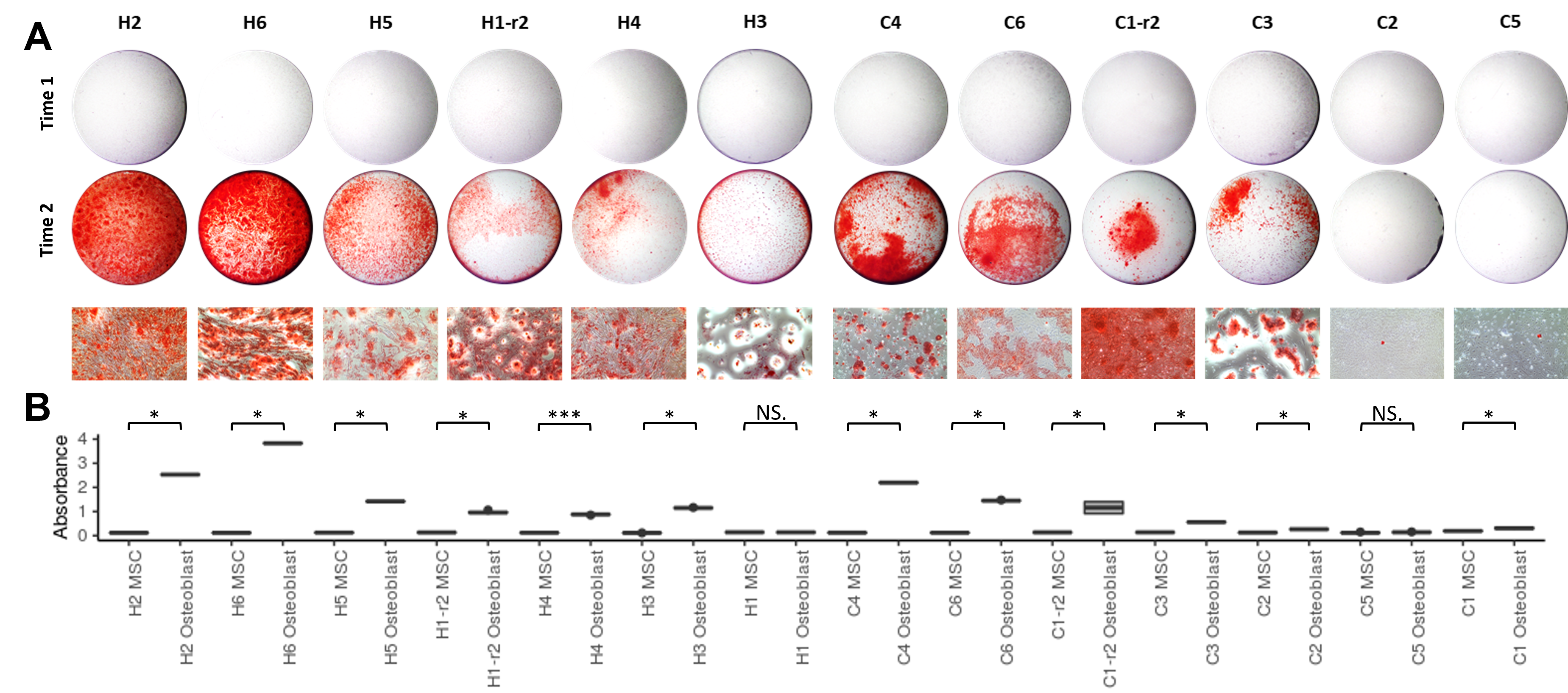

Supplement: S2 Fig — (A) Imaging of Alizarin Red stain in cell types from each cell line. Images in each row from top to bottom: (1) stained mesenchymal cells (Time 1) zoomed out to display the entire cell culture well, (2) stained osteogenic cells (Time 2) zoomed out to display the entire cell culture well, (3) phase contrast imaging at 4X of stained osteogenic cells (Time 2). (B) Box plots showing the absorbance values of extracted Alizarin Red stain from all biological and technical replicates. Statistical significance was determined using one-sided Mann Whitney tests. Box plots: middle line marks the median, box outlines the first and third quartiles, whiskers extend to 1.5 times the interquartile range. Significance: NS. p>0.05, * p<0.05, ** p<0.01, *** p <0.001. (TIF) [file pgen.1010073.s021.tif]

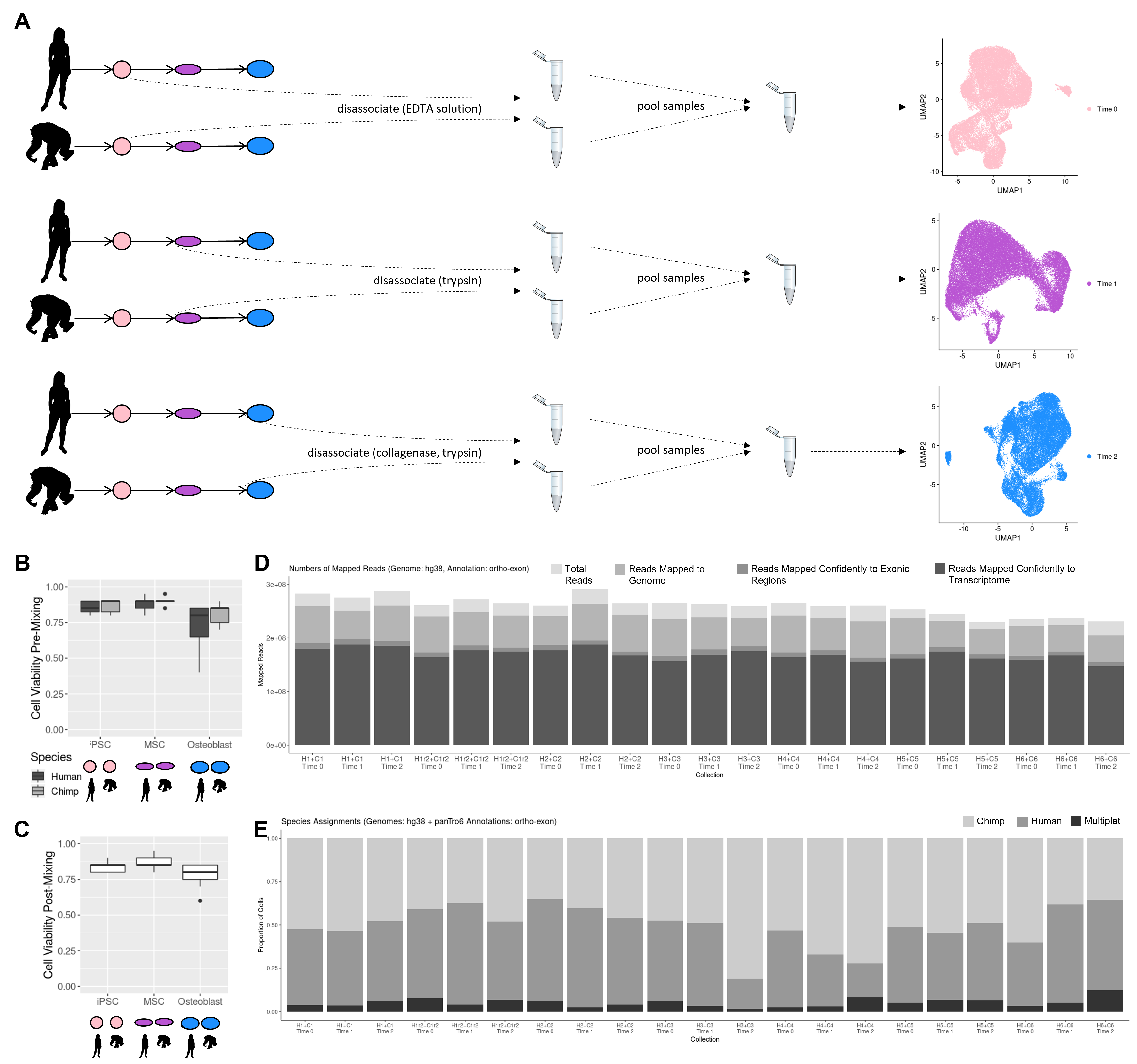

Supplement: S3 Fig — (A) Schematic of the dissociation and pooling protocol for 10X single-cell collections. (B) Box plots show cell viabilities for each species and cell type after disassociation. (C) Box plots show cell viabilities for pooled samples of human and chimpanzee cells after mixing. (D) Proportion of mapped scRNA-seq reads. (E) Proportions of species assignments for each 10X collection using Cell Ranger. Box plots: middle line marks the median, box outlines the first and third quartiles, whiskers extend to 1.5 times the interquartile range. Silhouette images were adapted from http://phylopic.org/ and courtesy of T. Michael Keesey and Tony Hisgett (http://creativecommons.org/licenses/by/3.0/). (TIF) [file pgen.1010073.s022.tif]

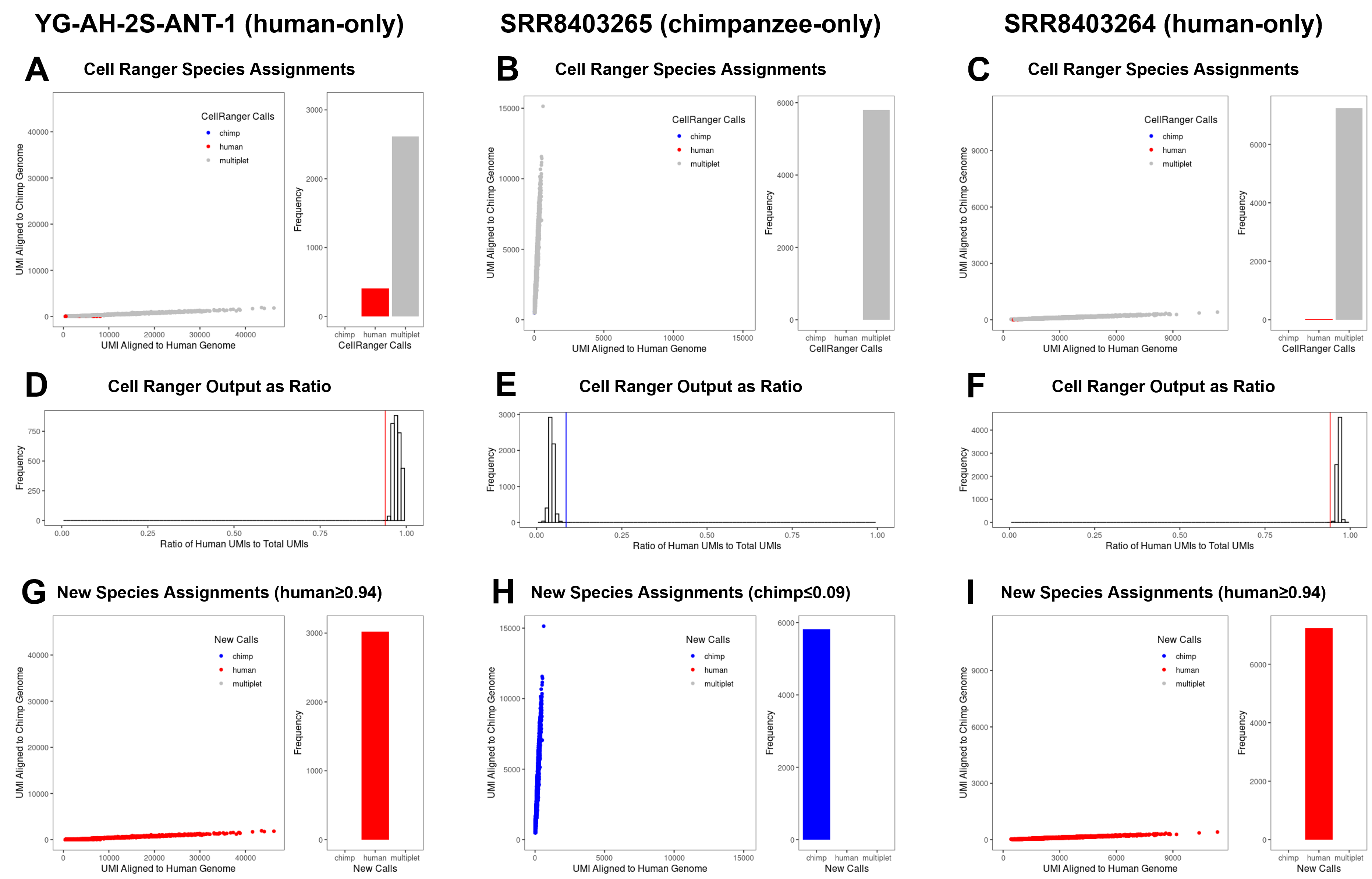

Supplement: S4 Fig — Results of running Cell Ranger on one dataset that contained only human cells [YG-AH-2S-ANT-1] (A,D,G), one dataset that contained only chimpanzee cells [SRR8403265] (B,E,H), and one dataset that contained only human cells [SRR8403264] (C,F,I). Top plots show species assignments for each cell when using the standard Cell Ranger pipeline (A,B,C). Plots in each set from left to right: (1) UMI counts aligned to the human genome vs. UMI counts aligned to the chimpanzee genome with cells colored by assignment, (2) number of cells called as human, chimpanzee, or multiplet. Middle plot shows a histogram of the ratio of human-aligned UMI counts per cells vs. the total number of aligned UMI counts per cells (bars near 0 are likely chimpanzee cells and bars near 1 are likely human cells) (D,E,F). Bottom plots show an alternative species assignment using low cutoffs (~0.9 for humans and ~0.1 for chimps) based on the ratios shown in the histograms (G,H,I). Plots in each set from left to right: (1) UMI counts aligned to the human genome vs. UMI counts aligned to the chimpanzee genome with cells colored by assignment, (2) number of cells called as human, chimpanzee, or multiplet. (TIF) [file pgen.1010073.s023.tif]

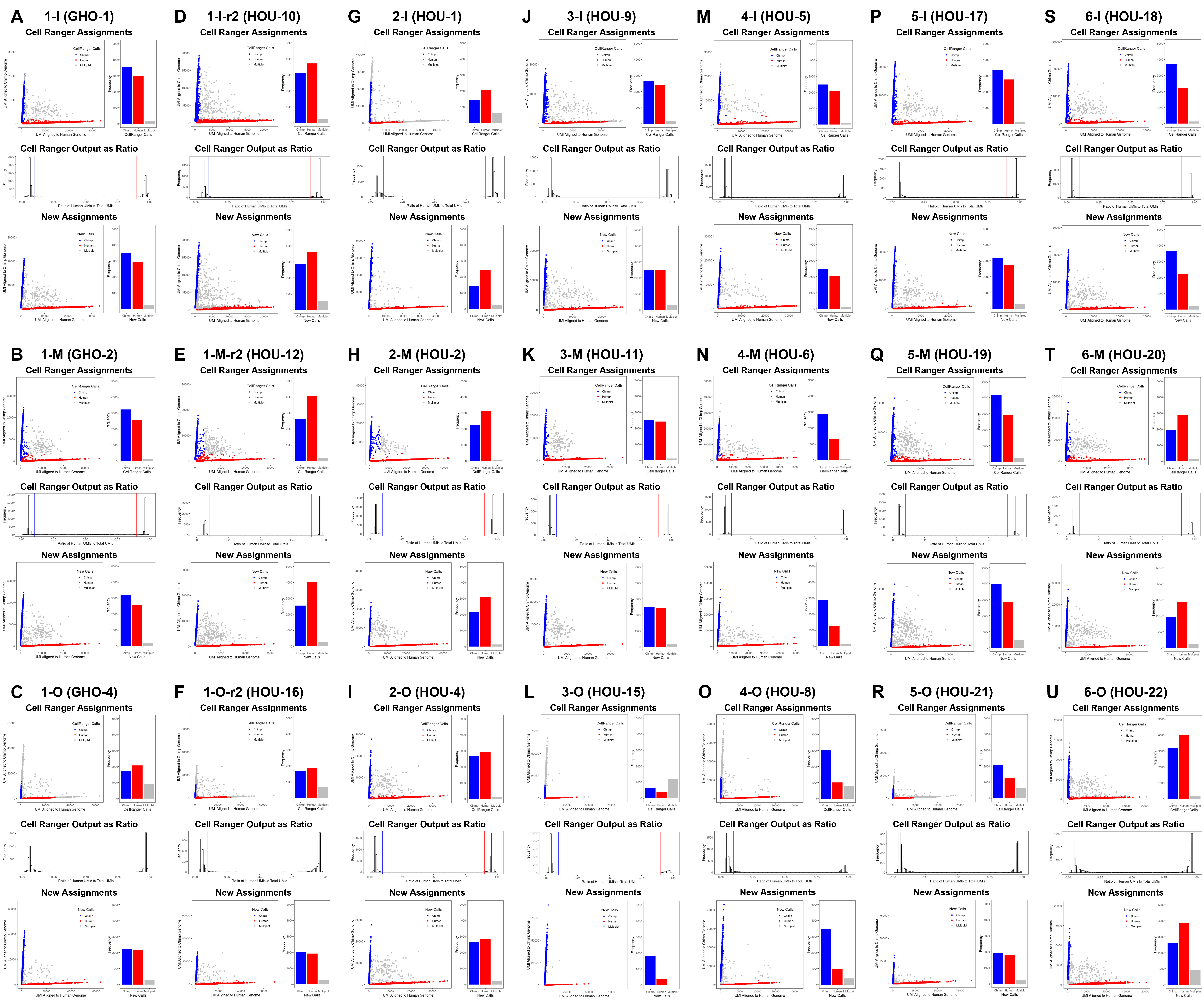

Supplement: S5 Fig — Results of running Cell Ranger vs. our modification on all datasets in the study. Top plots show species assignments for each cell when using the standard Cell Ranger pipeline (A,B,C,J,M,P,S). Plots in each set from left to right: (1) UMI counts aligned to the human genome vs. UMI counts aligned to the chimpanzee genome with cells colored by assignment, (2) number of cells called as human, chimpanzee, or multiplet. Middle plot shows a histogram of the ratio of human-aligned UMI counts per cells vs. the total number of aligned UMI counts per cells (bars near 0 are likely chimpanzee cells and bars near 1 are likely human cells) (D,E,F,K,N,Q,T). Bottom plots show an alternative species assignment using low cutoffs (~0.9 for humans and ~0.1 for chimps) based on the ratios shown in the histograms (G,H,I,L,O,R,U). Plots in each set from left to right: (1) UMI counts aligned to the human genome vs. UMI counts aligned to the chimpanzee genome with cells colored by assignment, (2) number of cells called as human, chimpanzee, or multiplet. (TIF) [file pgen.1010073.s024.tif]

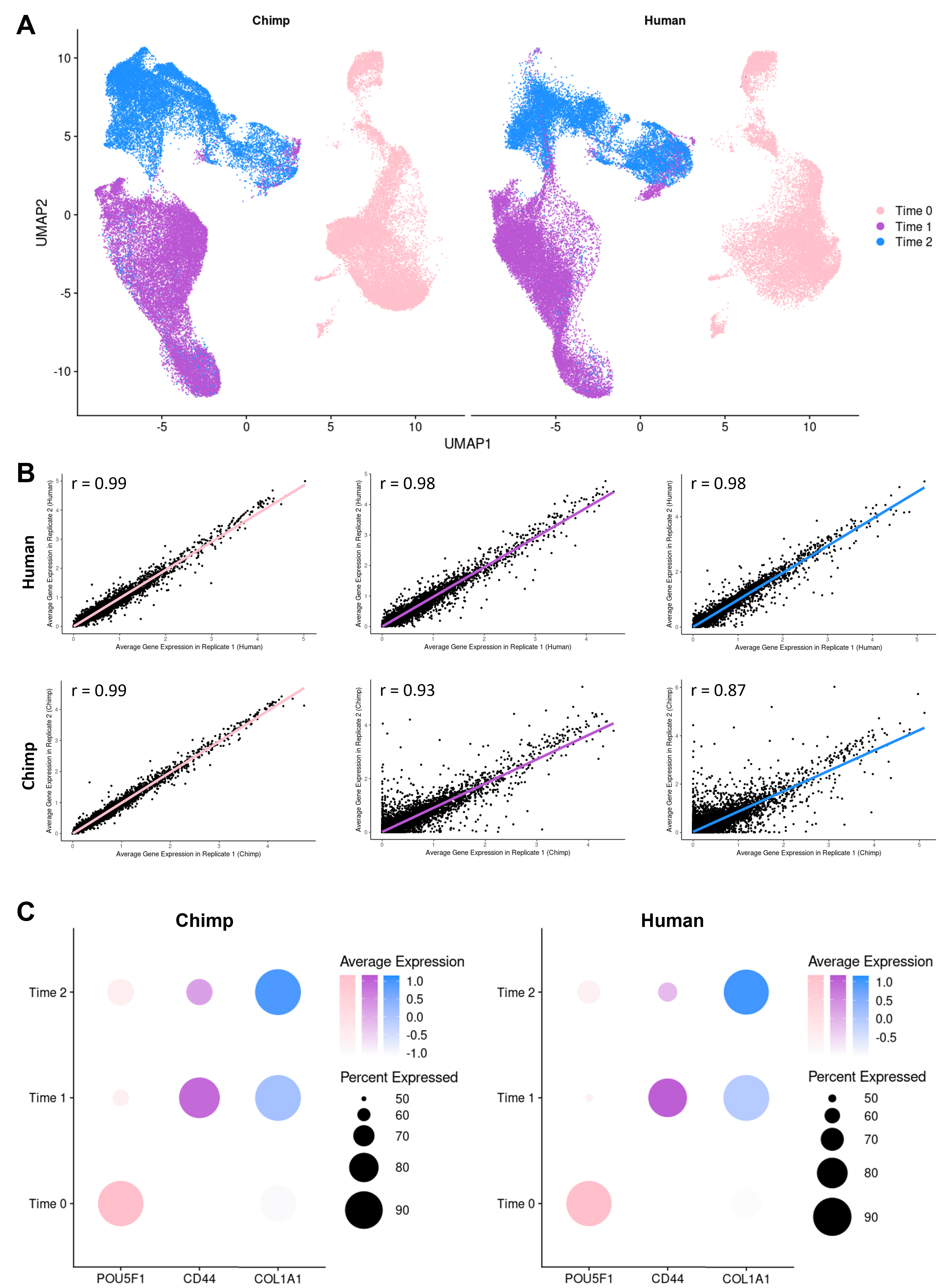

Supplement: S6 Fig — (A) UMAP dimensional reduction plots of scRNA-seq data with cells labeled by the stage of differentiation at which they were collected and separated by species. (B) The correlation of average gene expression patterns between technical replicates separated by species. Plots from left to right: (1) correlation in pluripotent cells (Time 0), (2) correlation in mesenchymal cells (Time 1), (3) correlation in osteogenic cells (Time 2). (C) Dot plots depicting the scaled average expression (dot color intensity) and the proportion of cells expressing each gene (dot size) of candidate genes (x-axis) at each stage of differentiation (y-axis). (TIF) [file pgen.1010073.s025.tif]

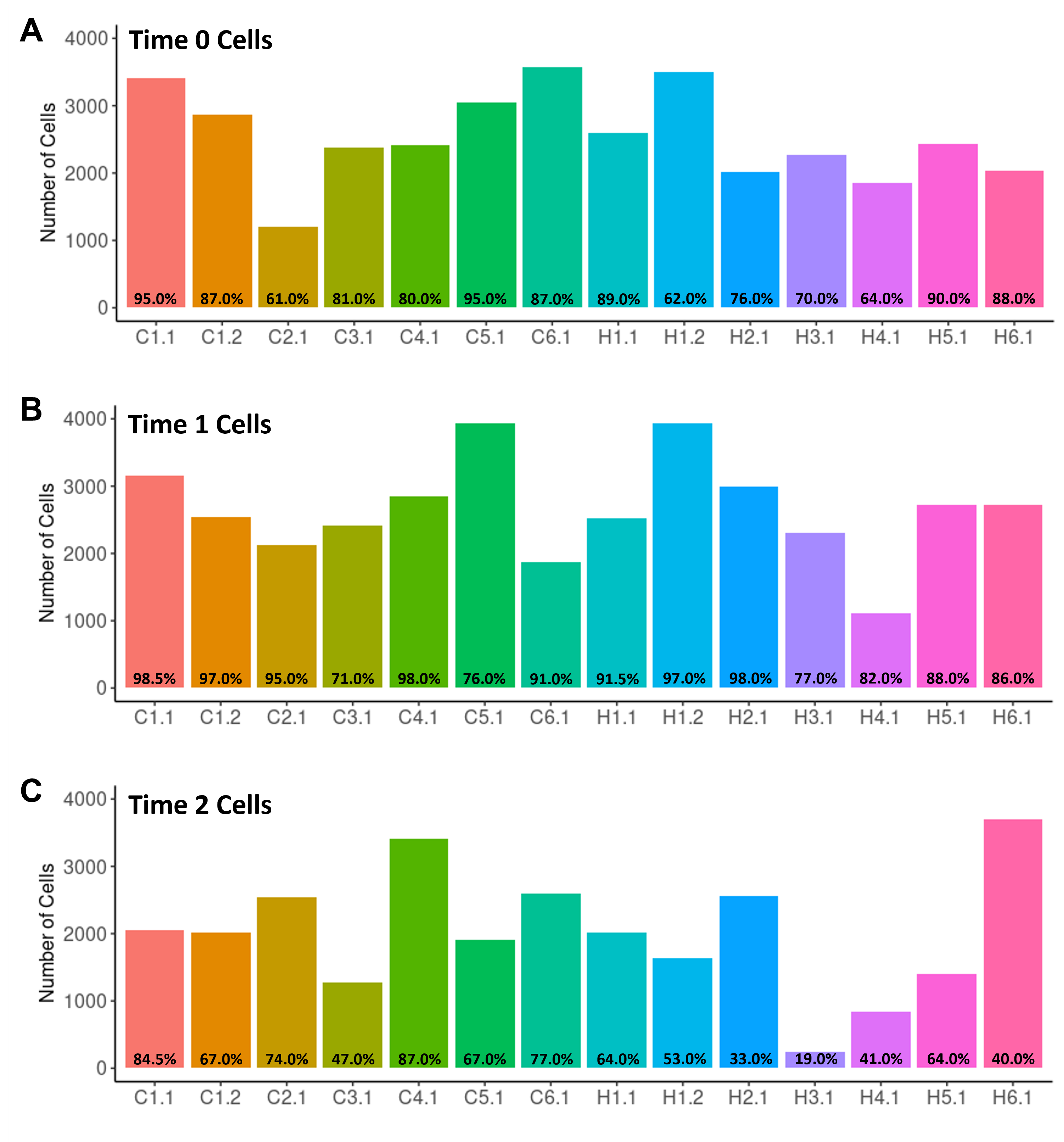

Supplement: S7 Fig — (A) Bar plot depicting the number of cells collected at pluripotent Time 0 from each cell line, with the average cell viability measure (post-dissociation, pre-sample-pooling) denoted within the corresponding bar. (B) Bar plot depicting the number of cells collected at mesenchymal Time 1 from each cell line, with the average cell viability measure denoted within the corresponding bar. (C) Bar plot depicting the number of cells collected at osteogenic Time 2 from each cell line, with the average cell viability measure denoted within the corresponding bar. (TIF) [file pgen.1010073.s026.tif]

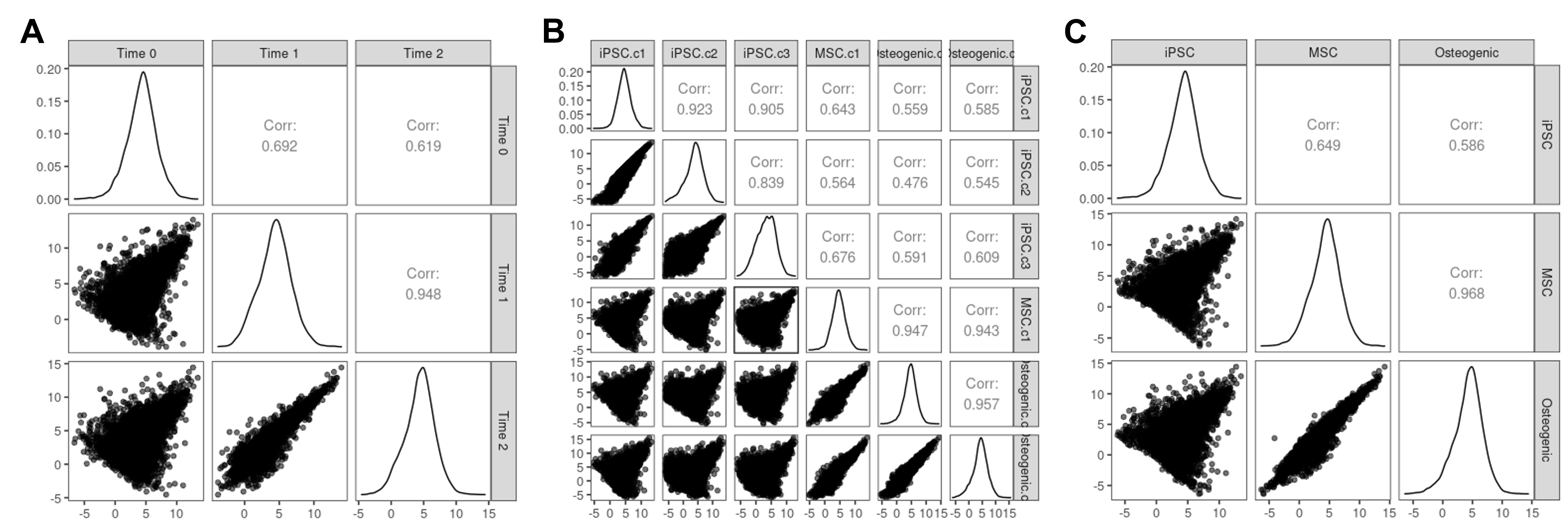

Supplement: S8 Fig — (A) Pairwise correlations of pseudobulk counts for each gene between stages of differentiation. (B) Pairwise correlations of pseudobulk counts for each gene between unsupervised clusters at a resolution of 0.05. (C) Pairwise correlations of pseudobulk counts for each gene between general ad hoc assignments. (TIF) [file pgen.1010073.s027.tif]

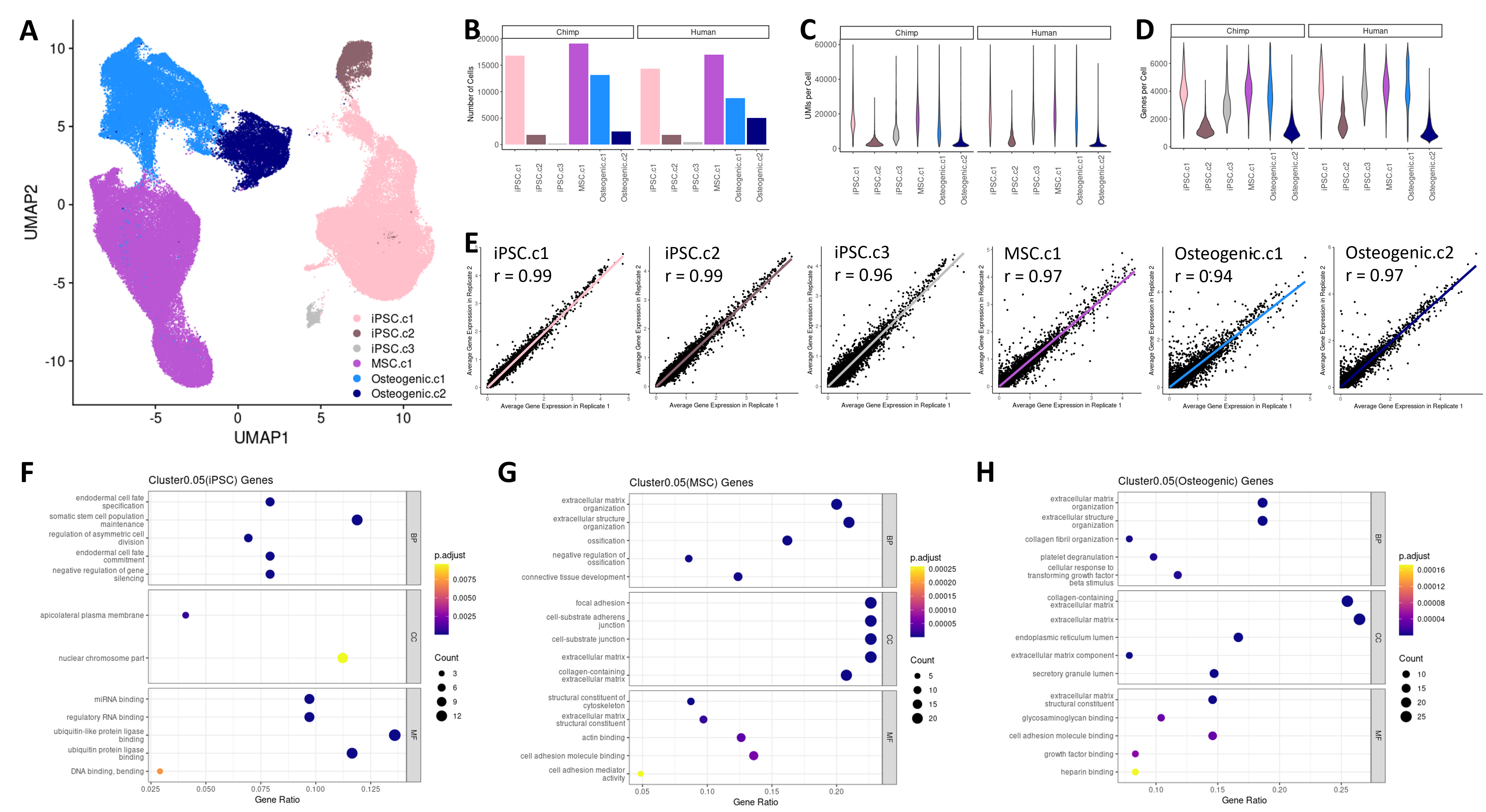

Supplement: S9 Fig — (A) UMAP dimensional reduction plot of scRNA-seq data with cells labeled by their assigned unsupervised cluster (resolution = 0.05). (B) Bar plot depicting the number of chimpanzee and human cells assigned to each cluster. (C) Violin plots displaying the distribution of UMI counts per cell for each cluster. (D) Violin plots displaying the distribution of gene counts per cell for each cluster. (E) The correlation of average gene expression patterns between technical replicates (human and chimpanzee) collected in each cluster. (F-H) Enrichment of GO functional categories in marker genes for iPSC.c1 (F), MSC.c1 (G), and Osteogenic.c1 (H). The top 5 GO functions identified in biological processes (BP), cell components (CC), and molecular functions (MF) are displayed along with the adjusted p-value (p-adjust), the number of marker genes overlapping a GO function (Count), and the ratio of overlapping to non-overlapping marker genes for a given GO function (Gene Ratio). (TIF) [file pgen.1010073.s028.tif]

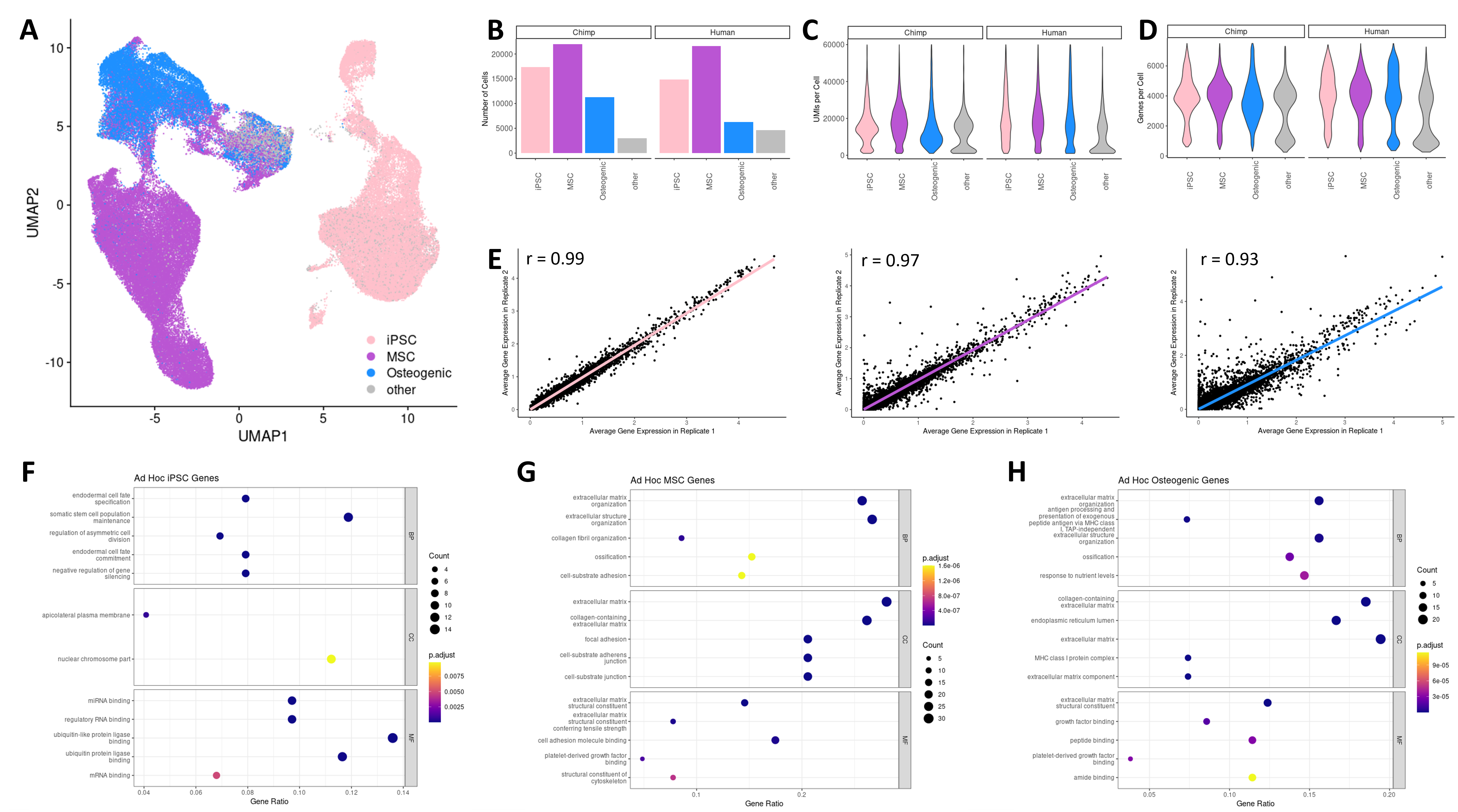

Supplement: S10 Fig — (A) UMAP dimensional reduction plot of scRNA-seq data with cells labeled by their ad hoc assignment. (B) Bar plot depicting the number of chimpanzee and human cells in each ad hoc assignment. (C) Violin plots displaying the distribution of UMI counts per cell for each ad hoc assignment. (D) Violin plots displaying the distribution of gene counts per cell for each ad hoc assignment. (E) The correlation of average gene expression patterns between technical replicates (human and chimpanzee) collected in each ad hoc assignment. (F-H) Enrichment of GO functional categories in marker genes for iPSC.c1 (F), MSC.c1 (G), and Osteogenic.c1 (H). The top 5 GO functions identified in biological processes (BP), cell components (CC), and molecular functions (MF) are displayed along with the adjusted p-value (p-adjust), the number of marker genes overlapping a GO function (Count), and the ratio of overlapping to non-overlapping marker genes for a given GO function (Gene Ratio). (TIF) [file pgen.1010073.s029.tif]

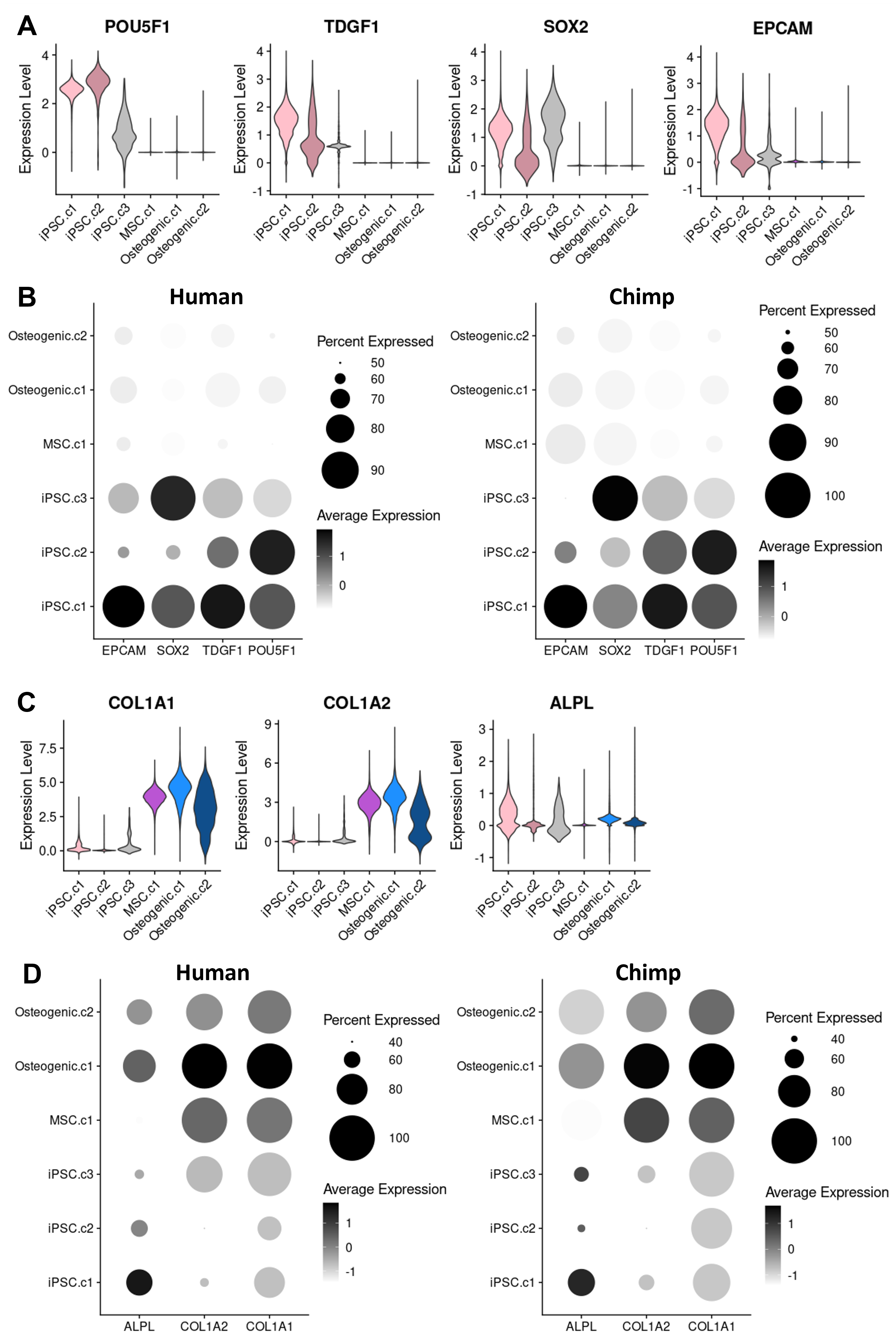

Supplement: S11 Fig — (A) Violin plots displaying the distribution of pluripotent marker gene expression levels (POU5F1, TDGF1, SOX2, EPCAM) in each unsupervised cluster (resolution = 0.05). (B) Dot plots depicting the scaled average expression (dot color intensity) and the proportion of cells expressing each gene (dot size) of pluripotent marker genes (x-axis) in each cluster (y-axis). (C) Violin plots displaying the distribution of osteogenic marker gene expression levels (COL1A1, COL1A2, ALPL) in each cluster. (D) Dot plots depicting the scaled average expression (dot color intensity) and the proportion of cells expressing each gene (dot size) of osteogenic marker genes (x-axis) in each cluster (y-axis). (TIF) [file pgen.1010073.s030.tif]

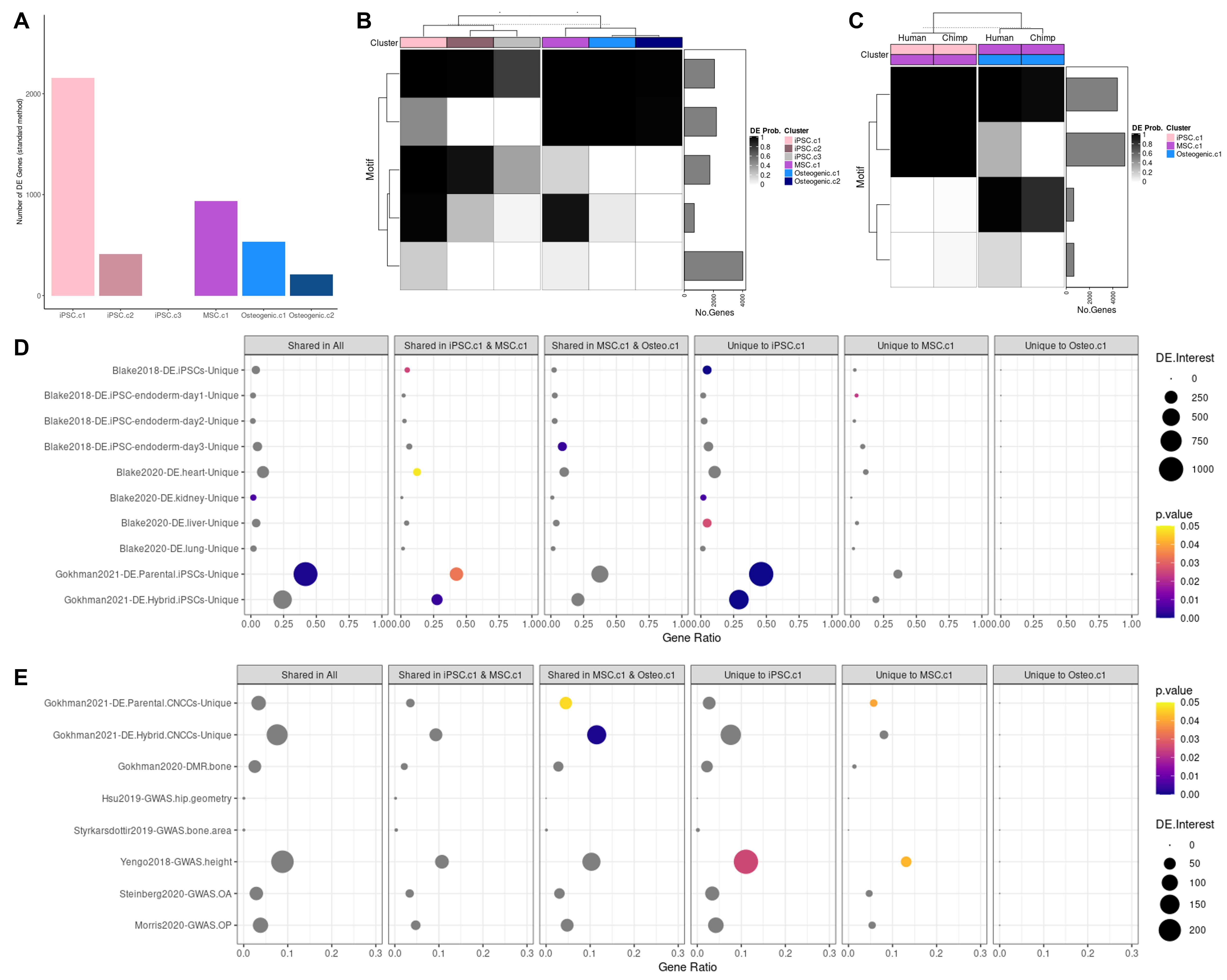

Supplement: S12 Fig — (A) Bar plot showing the number of interspecific DE genes identified for each unsupervised cluster (resolution = 0.05) using standard methods. (B-C) Correlation motifs based on the probability of differential expression between species for each cluster (B) and correlation motifs based on the probability of differential expression across clusters (iPSC.c1, MSC.c1, Osteogenic.c1) for each species (C) with the number of genes assigned to each motif shown in the bar plot on the right and the posterior probability that a gene is DE between two clusters in a given species shown by the shading of each box. (D-E) Enrichment of external DE gene sets among Cormotif interspecific DE genes identified for each cluster with the p-value (p.value), the number of DE genes overlapping an external gene set (DE.Interest), and the ratio of overlapping to non-overlapping DE genes for a given external gene set (Gene Ratio) denoted. (TIF) [file pgen.1010073.s031.tif]

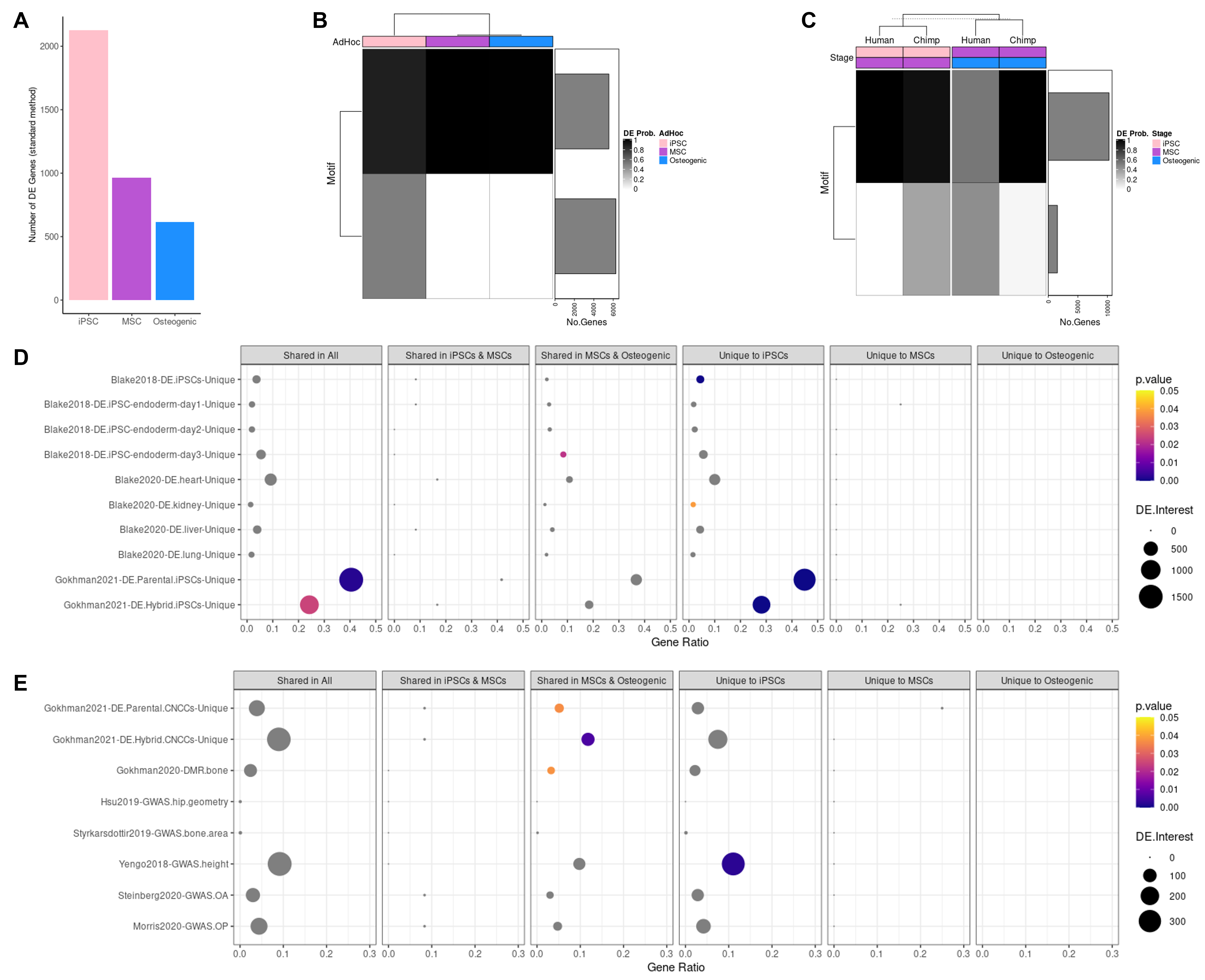

Supplement: S13 Fig — (A) Bar plot showing the number of standard interspecific DE genes identified for each ad hoc assignment. (B-C) Correlation motifs based on the probability of differential expression between species for each ad hoc assignment (B) and correlation motifs based on the probability of differential expression across ad hoc assignments for each species (C) with the number of genes assigned to each motif shown in the bar plot on the right and the posterior probability that a gene is DE between two clusters in a given species shown by the shading of each box. (D-E) Enrichment of external DE gene sets among Cormotif interspecific DE genes identified for each ad hoc assignment with the p-value (p.value), the number of DE genes overlapping an external gene set (DE.Interest), and the ratio of overlapping to non-overlapping DE genes for a given external gene set (Gene Ratio) denoted. (TIF) [file pgen.1010073.s032.tif]

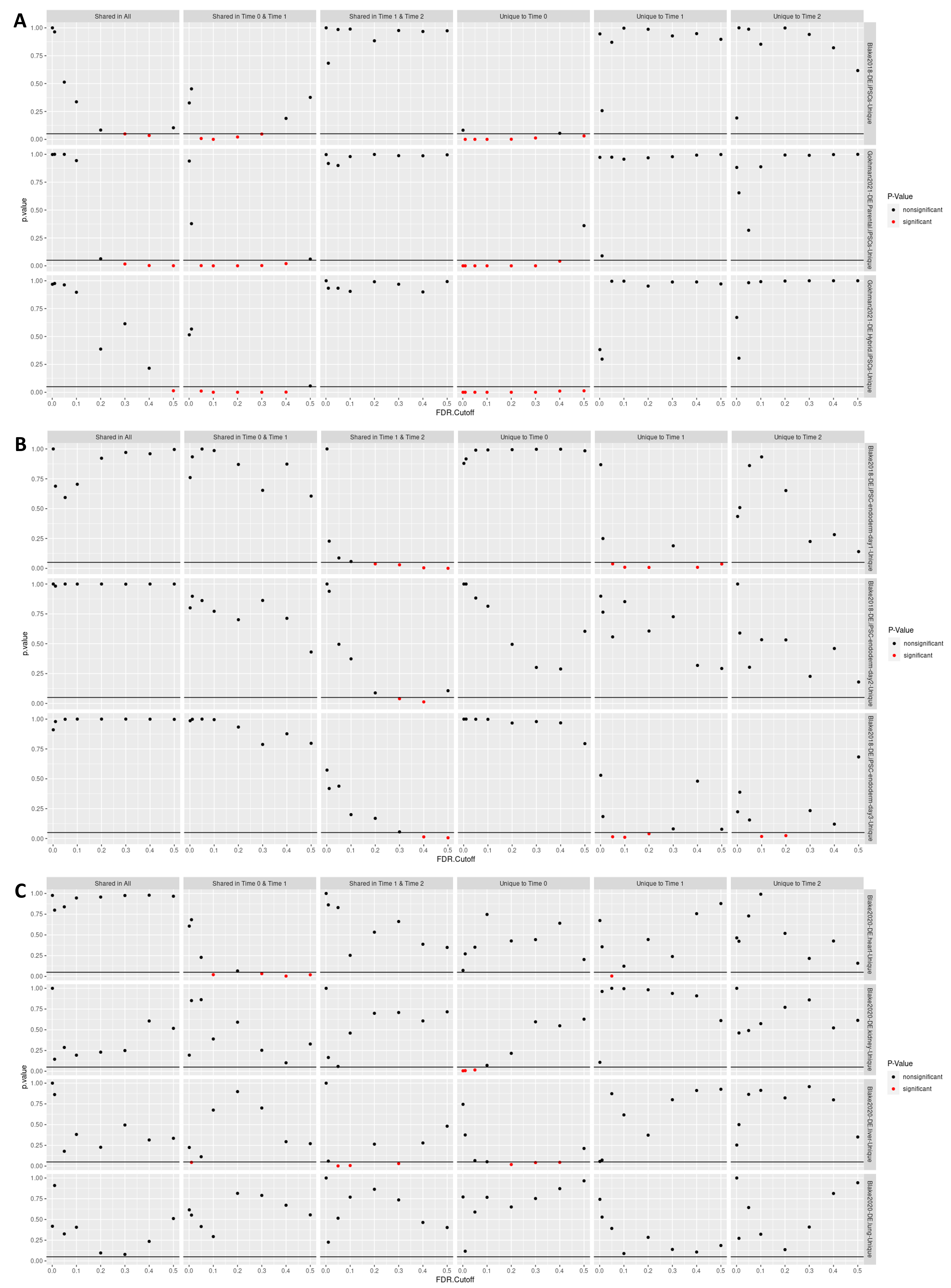

Supplement: S14 Fig — Enrichment p-values (p.value) of external DE gene sets among interspecific DE genes identified across stages of differentiation using standard methods and a range of different FDR cutoffs (FDR.Cutoff). A p-value of 0.05 is denoted by a horizontal line on each plot, and significant enrichments (p-value < 0.05) are highlighted in red. External DE gene sets were chosen for validation purposes–(A) previously identified interspecific DE genes in iPSCs are expected to only be enriched in interspecific DE genes unique to pluripotent cells (Time 0), (B) previously identified interspecific DE genes in alternative cell types (non-pluripotent, non-mesenchymal, and non-osteogenic) are not expected to be enriched in any interspecific DE genes identified in this study, and (C) previously identified interspecific DE genes in alternative tissue types (non-pluripotent, non-mesenchymal, and non-osteogenic) are not expected to be enriched in any interspecific DE genes identified in this study. (TIF) [file pgen.1010073.s033.tif]

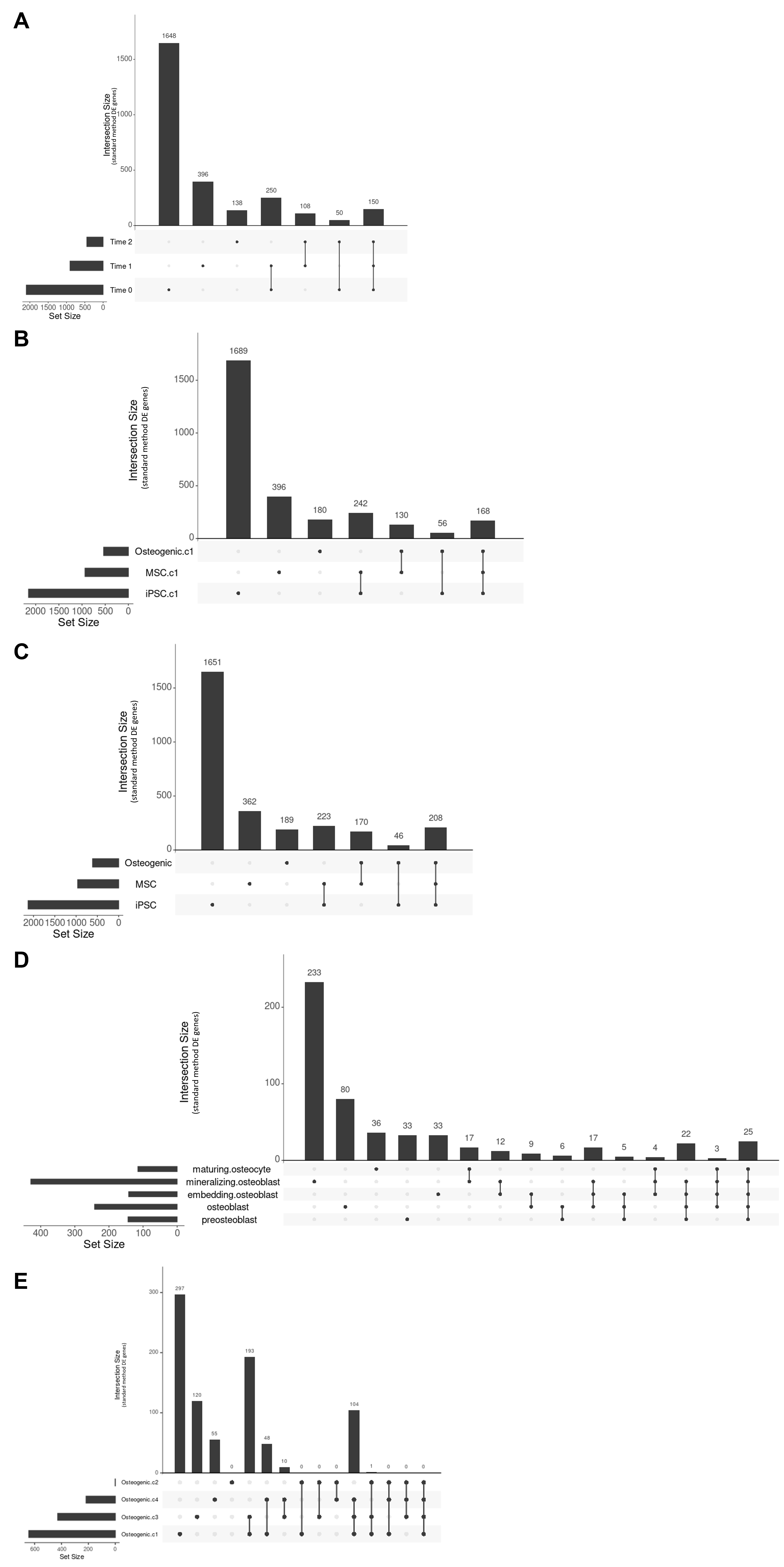

Supplement: S15 Fig — (A) Details regarding the intersection of standard interspecific DE genes identified across stages of differentiation. (B) Details regarding the intersection of standard interspecific DE genes identified across general unsupervised clusters (resolution = 0.05). (C) Details regarding the intersection of standard interspecific DE genes identified across general ad hoc assignments. (D) Details regarding the intersection of standard interspecific DE genes identified across osteogenic ad hoc assignments. (E) Details regarding the intersection of standard interspecific DE genes identified across osteogenic unsupervised clusters (resolution = 0.50). (TIF) [file pgen.1010073.s034.tif]

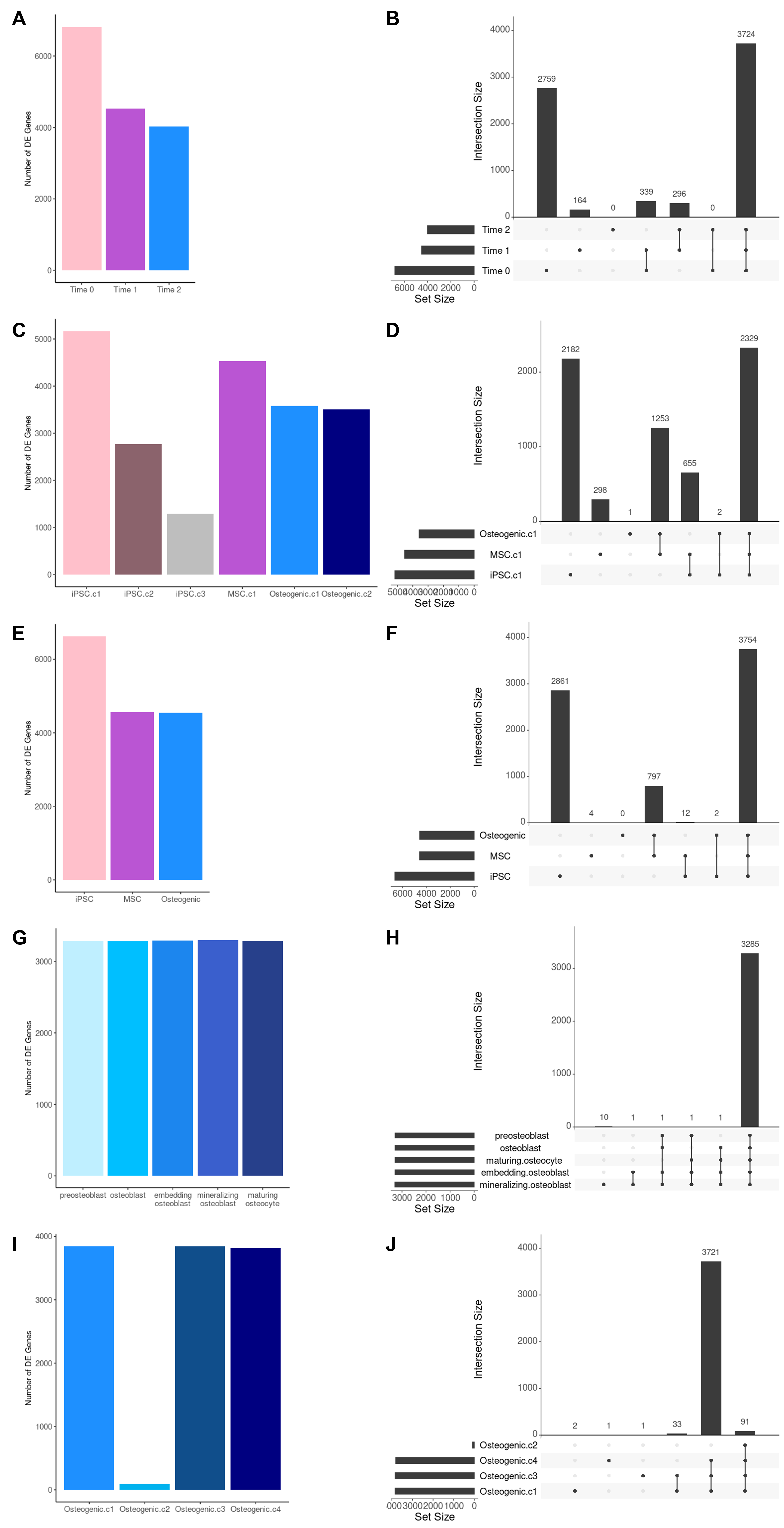

Supplement: S16 Fig — (A-B) Plots describing Cormotif interspecific DE genes identified across stages of differentiation. (C-D) Plots describing Cormotif interspecific DE genes identified across general unsupervised clusters (resolution = 0.05). (E-F) Plots describing Cormotif interspecific DE genes identified across general ad hoc assignments. (G-H) Plots describing Cormotif interspecific DE genes identified across osteogenic ad hoc assignments. (I-J) Plots describing Cormotif interspecific DE genes identified across osteogenic unsupervised clusters (resolution = 0.50). Plots within each set from left to right: (1) bar plot showing the number of interspecific DE genes identified using Cormotif for given cell classifications (A,C,E,G,I), (2) details regarding the intersection of DE genes for given cell classification (B,D,F,H,J). (TIF) [file pgen.1010073.s035.tif]

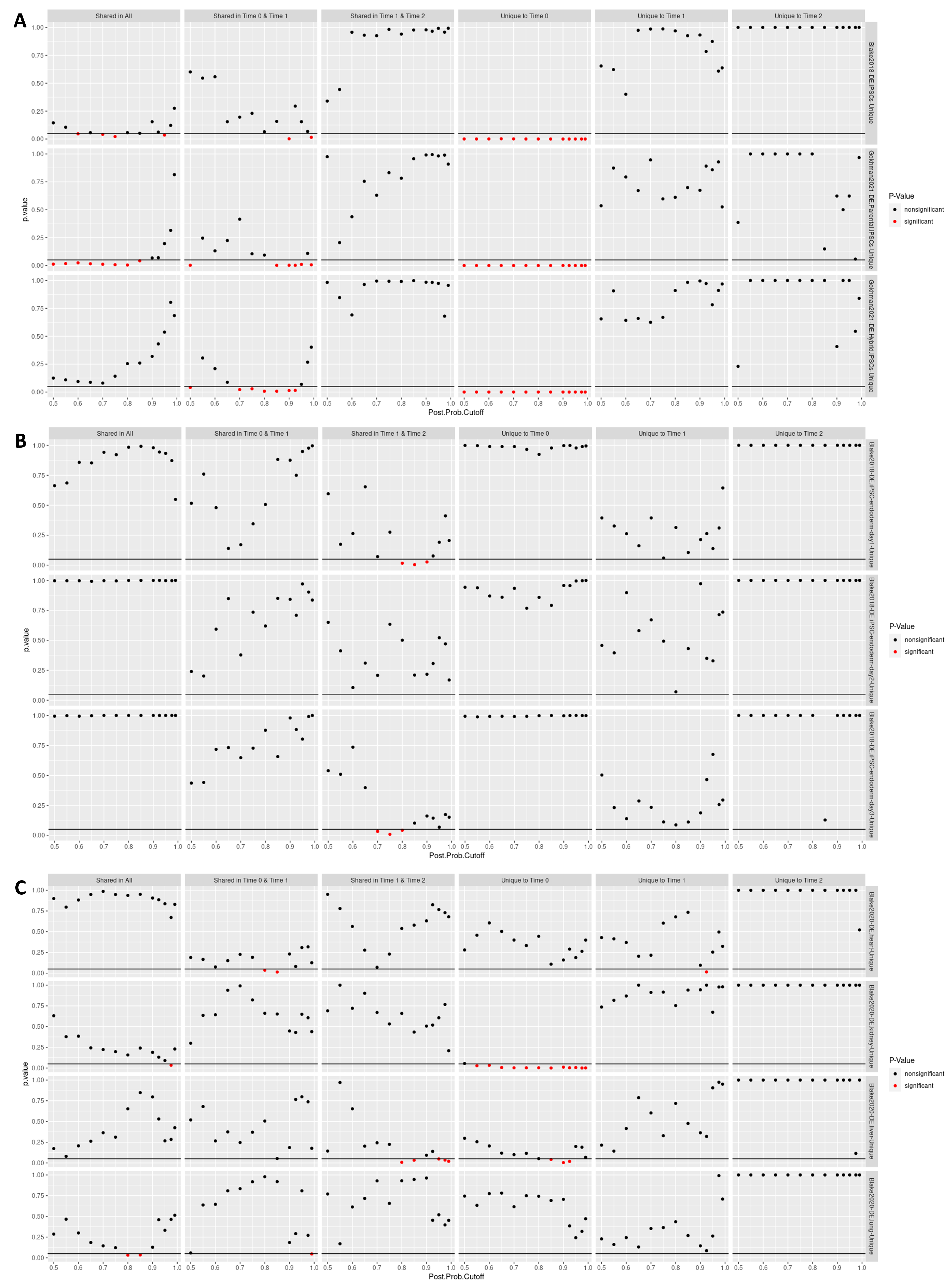

Supplement: S17 Fig — Enrichment p-values (p.value) of external DE gene sets among Cormotif interspecific DE genes identified across stages of differentiation using a range of different posterior probability cutoffs (Post. Prob. Cutoff). A p-value of 0.05 is denoted by a horizontal line on each plot, and significant enrichments (p-value < 0.05) are highlighted in red. External DE gene sets were chosen for validation purposes–(A) previously identified interspecific DE genes in iPSCs are expected to only be enriched in interspecific DE genes unique to pluripotent cells (Time 0), (B) previously identified interspecific DE genes in alternative cell types (non-pluripotent, non-mesenchymal, and non-osteogenic) are not expected to be enriched in any interspecific DE genes identified in this study, and (C) previously identified interspecific DE genes in alternative tissue types (non-pluripotent, non-mesenchymal, and non-osteogenic) are not expected to be enriched in any interspecific DE genes identified in this study. (TIF) [file pgen.1010073.s036.tif]

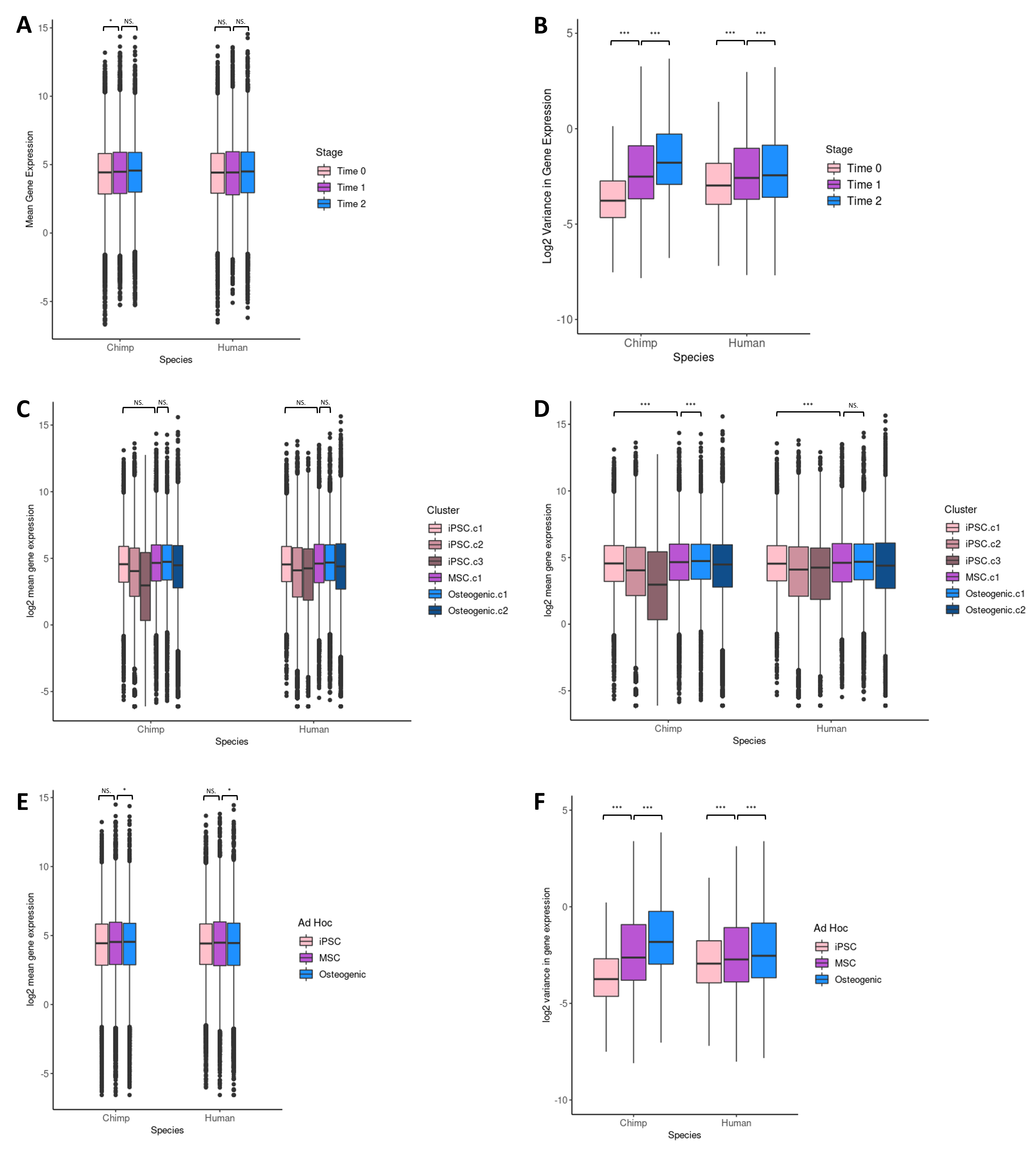

Supplement: S18 Fig — (A,C,E) Box plots of the mean gene expression values for given cell classifications in each species. (B,D,F) Box plots of the log2 transformed gene expression variance values for given cell classifications in each species. Statistical significance was determined using two-sided t-tests. (A-B) Mean and variance values for stages of differentiation. (C-D) Mean and variance values for general unsupervised clusters (resolution = 0.05). (E-F) Mean and variance values for general ad hoc assignments. Box plots: middle line marks the median, box outlines the first and third quartiles, whiskers extend to 1.5 times the interquartile range. Significance: NS. p>0.05, * p<0.05, ** p<0.01, *** p <0.001. (TIF) [file pgen.1010073.s037.tif]

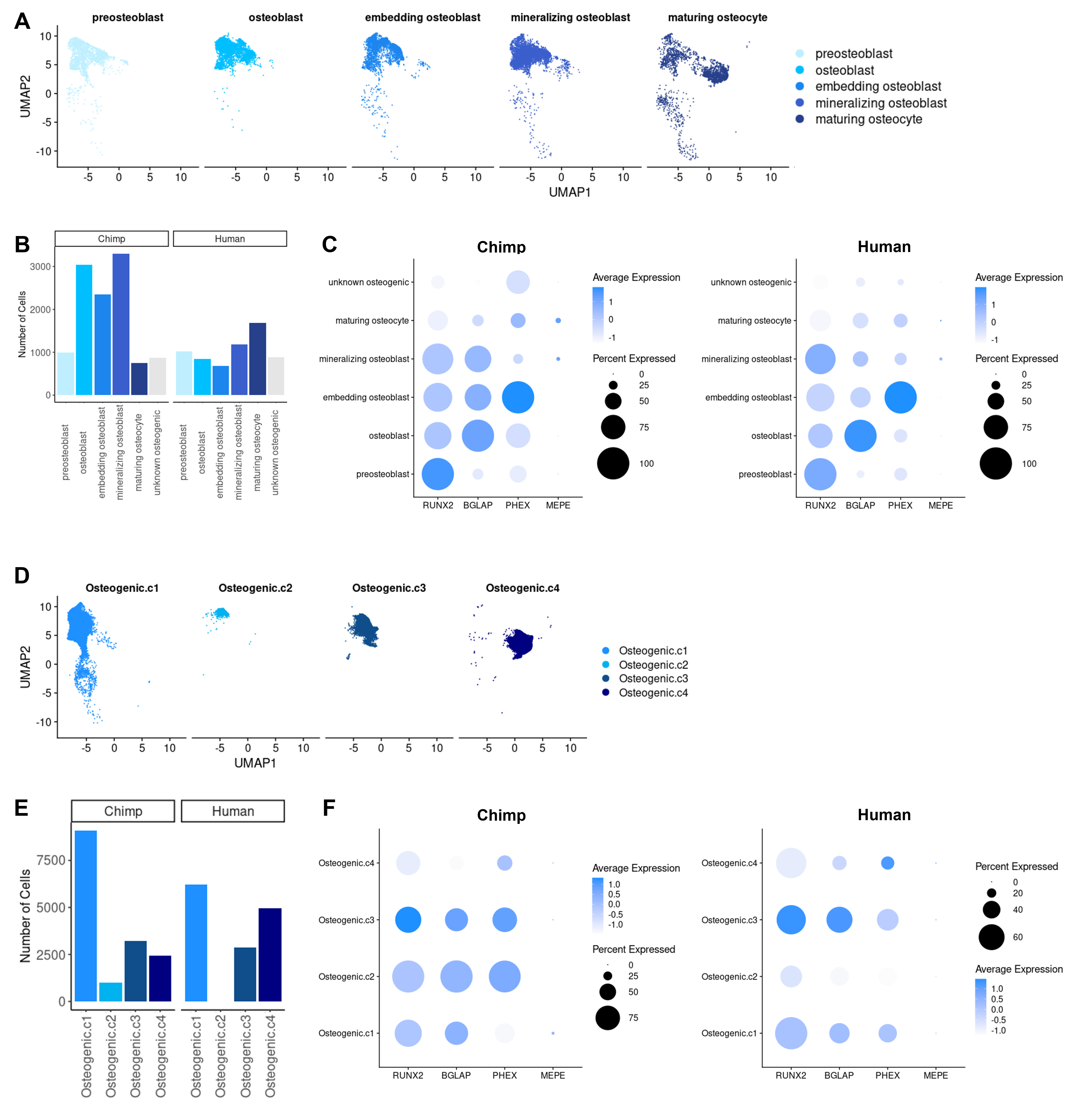

Supplement: S19 Fig — Plots compare (A-C) the osteogenic ad hoc assignment method to (D-F) the osteogenic unsupervised cluster method (resolution = 0.50). (A) UMAP dimensional reduction plots of scRNA-seq data with cells labeled by the stage of osteogenesis to which they were classified via the osteogenic ad hoc assignment method. (B) Bar plot depicting the number of chimpanzee and human cells for each osteogenic ad hoc assignment. (C) Dot plots depicting the scaled average expression (dot color intensity) and the proportion of cells expressing each gene (dot size) of candidate genes (x-axis) for each osteogenic ad hoc assignment (y-axis). (D) UMAP dimensional reduction plots of scRNA-seq data with cells labeled by the osteogenic unsupervised cluster to which they were assigned. (E) Bar plot depicting the number of chimpanzee and human cells for each osteogenic cluster. (F) Dot plots depicting the scaled average expression (dot color intensity) and the proportion of cells expressing each gene (dot size) of candidate genes (x-axis) for each osteogenic cluster (y-axis). (TIF) [file pgen.1010073.s038.tif]

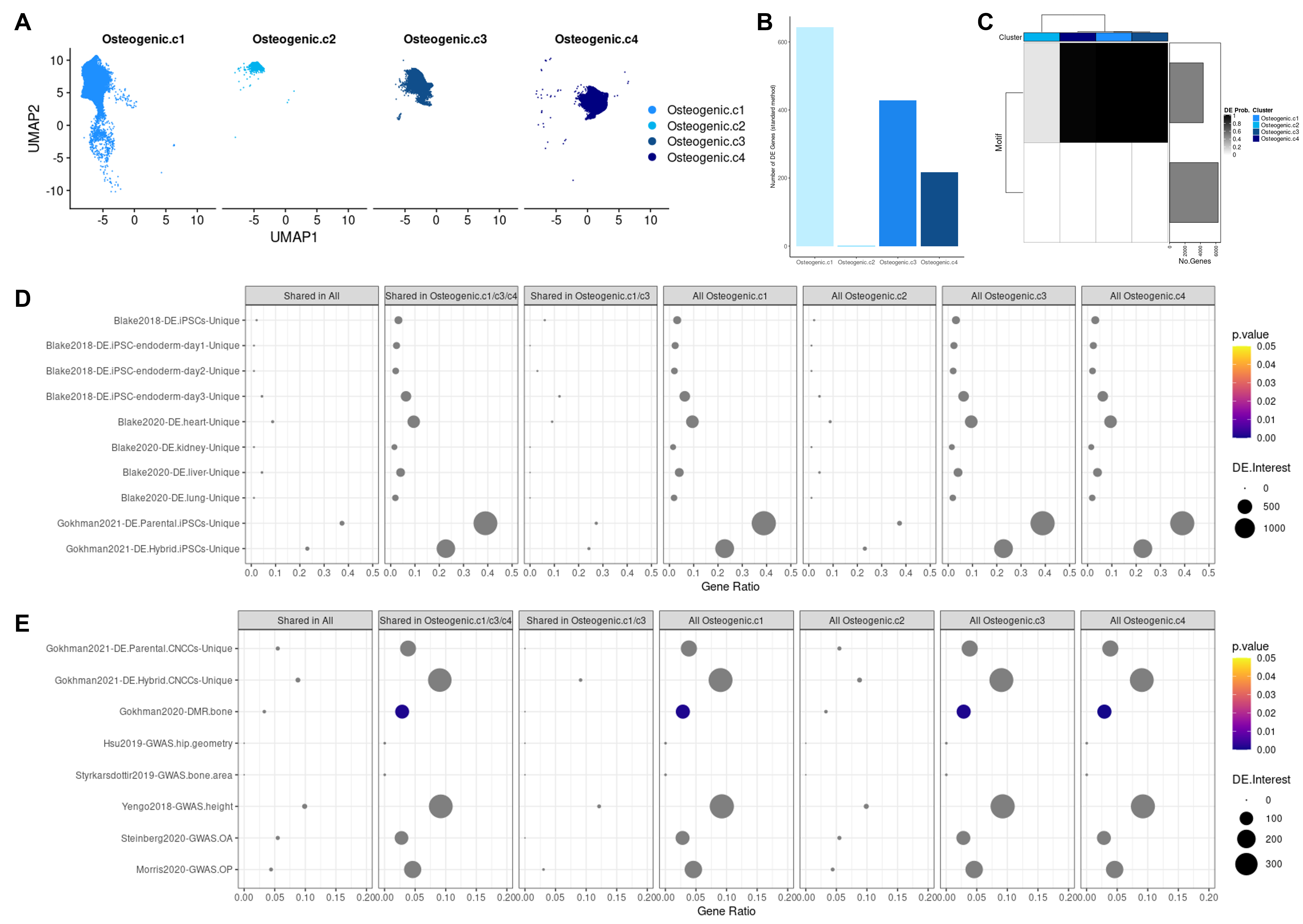

Supplement: S20 Fig — (A) UMAP dimensional reduction plot of scRNA-seq data with cells labeled by the osteogenic unsupervised cluster (resolution = 0.50) to which they were assigned. (B) Bar plot showing the number of interspecific DE genes identified for each osteogenic cluster using standard methods. (C) Correlation motifs based on the probability of differential expression between species for each osteogenic cluster with the number of genes assigned to each motif shown in the bar plot on the right and the posterior probability that a gene is DE shown by the shading of each box. (D-E) Enrichment of external DE gene sets among Cormotif interspecific DE genes identified for each osteogenic cluster with the p-value (p.value), the number of DE genes overlapping an external gene set (DE.Interest), and the ratio of overlapping to non-overlapping DE genes for a given external gene set (Gene Ratio) denoted. (TIF) [file pgen.1010073.s039.tif]

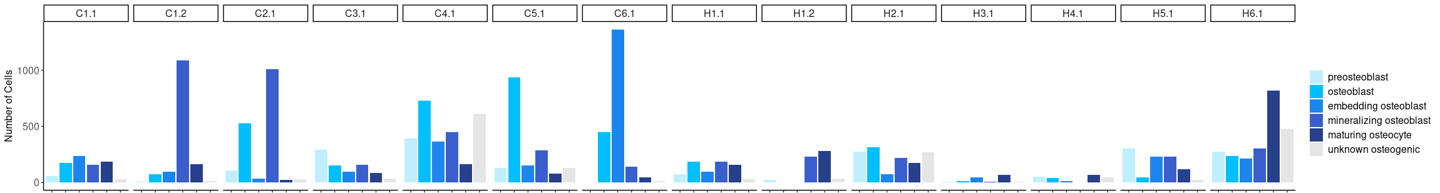

Supplement: S21 Fig — Bar plots depicting the number of osteogenic ad hoc assignment cells for each individual and replicate. (TIF) [file pgen.1010073.s040.tif]

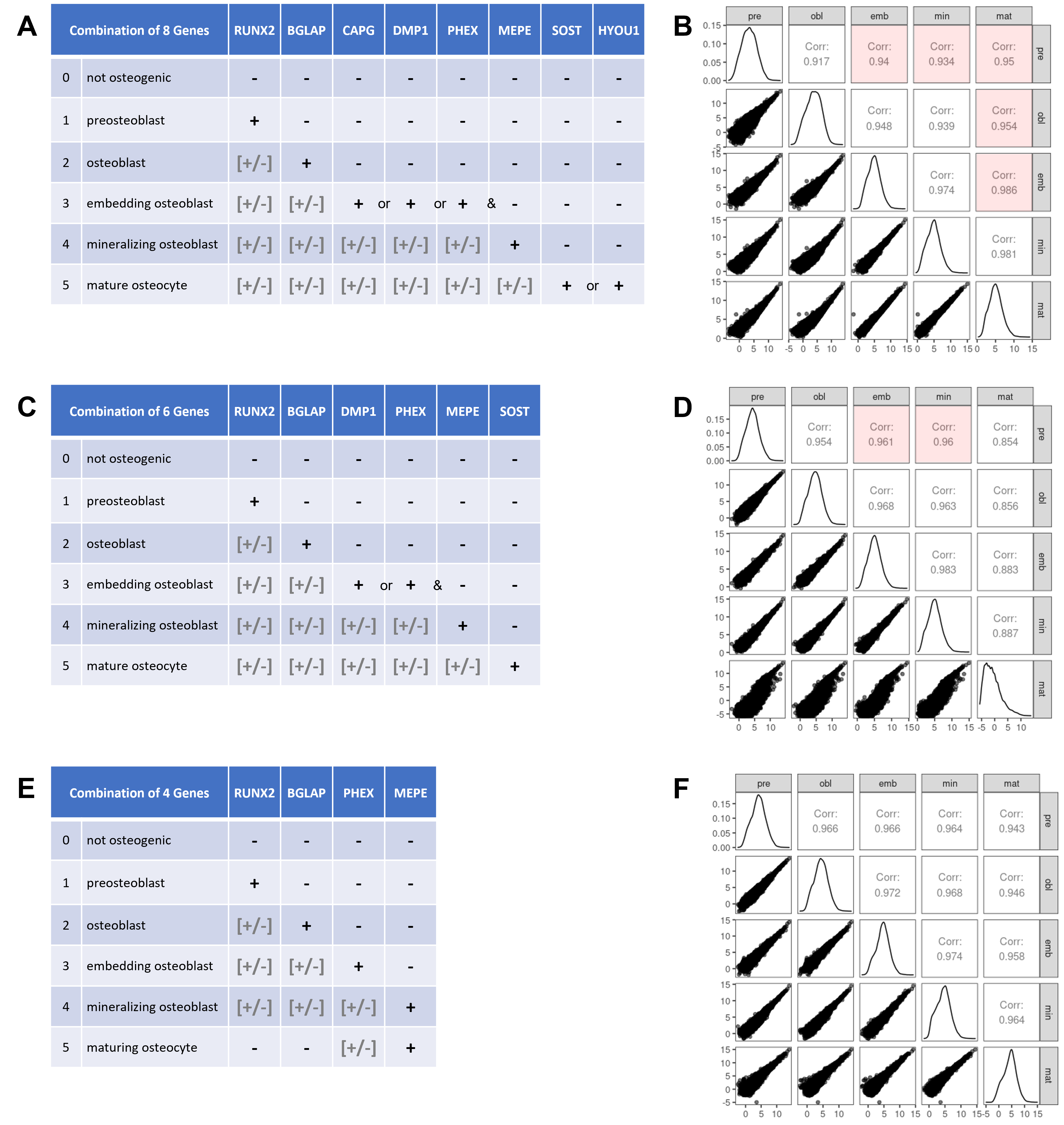

Supplement: S22 Fig — (A,C,E) Schematic for each considered osteogenic ad hoc assignment method and (B,D,F) pairwise correlations of pseudobulk counts for each gene between osteogenic ad hoc assignments. Correlation values highlighted in red indicate a deviation from the expected pattern. (A-B) Plots of the osteogenic ad hoc assignment method using 8 candidate genes. (C-D) Plots of the osteogenic ad hoc assignment method using 6 candidate genes. (E-F) Plots of the osteogenic ad hoc assignment method using 4 candidate genes. (TIF) [file pgen.1010073.s041.tif]

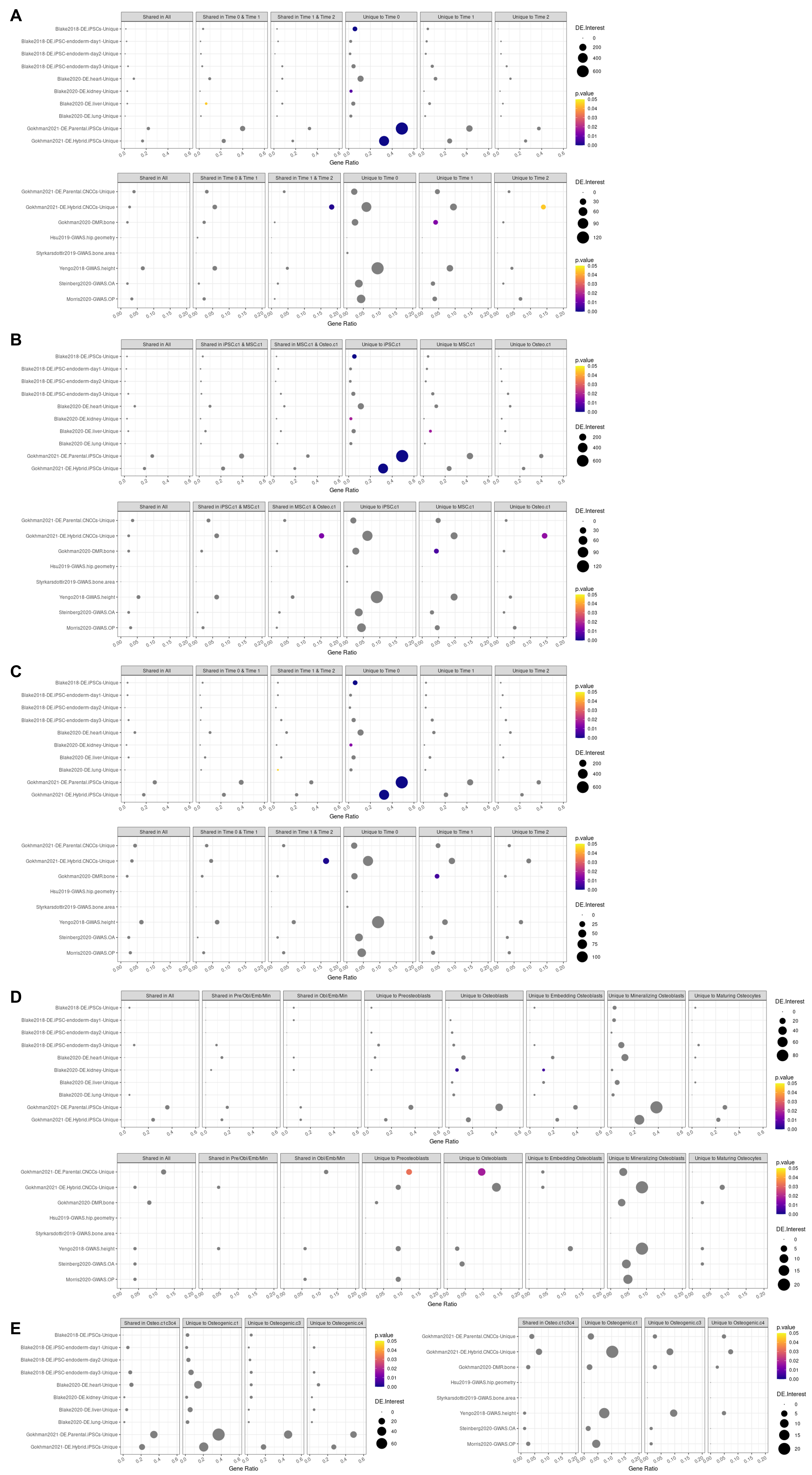

Supplement: S23 Fig — Enrichment of external DE gene sets among standard interspecific DE genes (FDR<0.01) identified for given cell classifications with the p-value (p.value), the number of DE genes overlapping an external gene set (DE.Interest), and the ratio of overlapping to non-overlapping DE genes for a given external gene set (Gene Ratio) denoted. (A) Enrichments across stages of differentiation. (B) Enrichments across general unsupervised clusters (resolution = 0.05). (C) Enrichments across general ad hoc assignments. (D) Enrichments across osteogenic ad hoc assignments. (E) Enrichments across osteogenic unsupervised clusters (resolution = 0.50). (TIF) [file pgen.1010073.s042.tif]

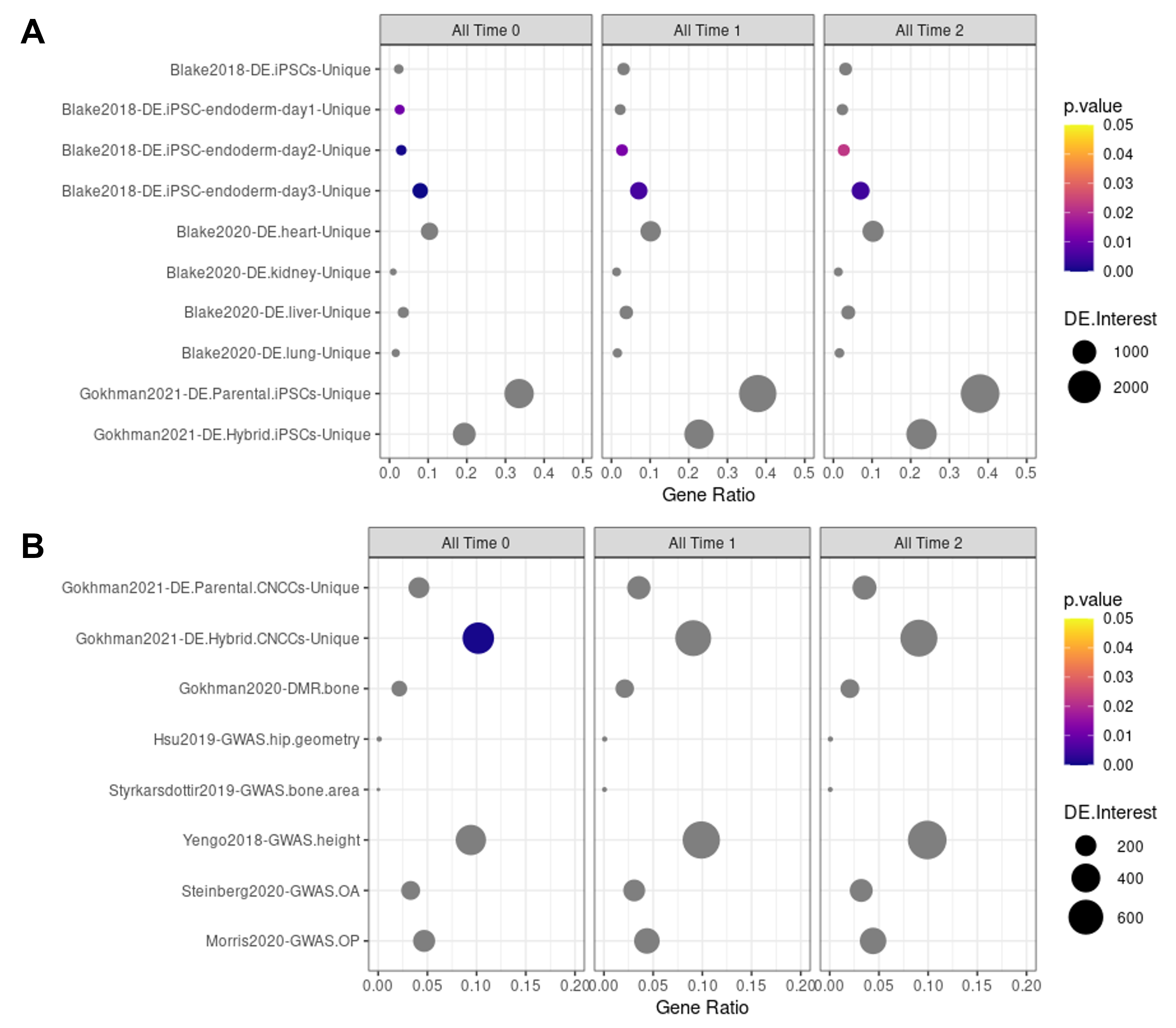

Supplement: S24 Fig — (A-B) Enrichment of external DE gene sets among Cormotif interspecific DE genes identified for each stage of differentiation for validation (A) and functional interpretation (B) with the p-value (p.value), the number of DE genes overlapping an external gene set (DE.Interest), and the ratio of overlapping to non-overlapping DE genes for a given external gene set (Gene Ratio) denoted. (TIF) [file pgen.1010073.s043.tif]

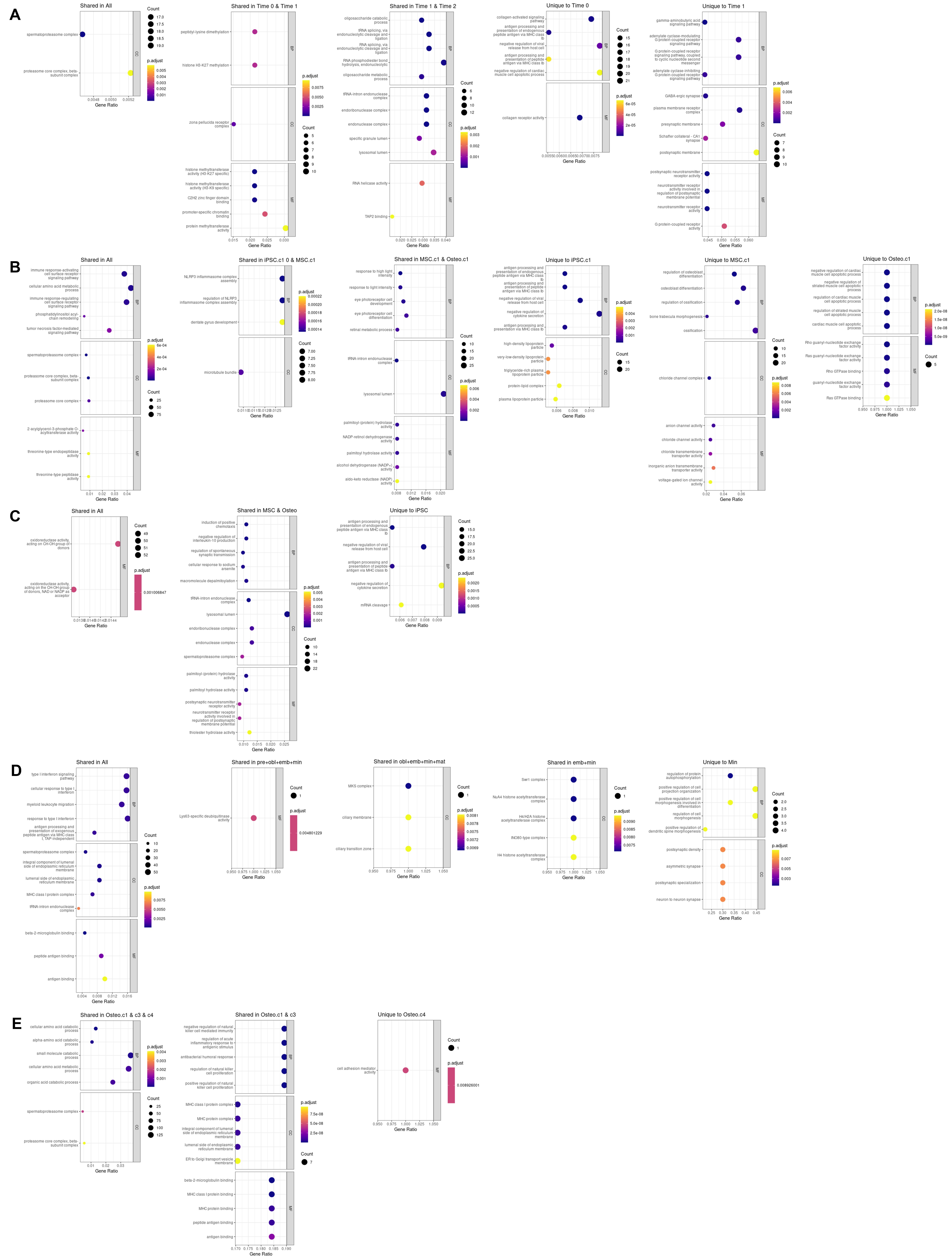

Supplement: S25 Fig — Enrichment of GO functional categories among Cormotif interspecific DE genes identified for a given cell classification. The top 5 GO functions identified in biological processes (BP), cell components (CC), and molecular functions (MF) are displayed along with the adjusted p-value (p-adjust), the number of marker genes overlapping a GO function (Count), and the ratio of overlapping to non-overlapping marker genes for a given GO function (Gene Ratio). (A) Enrichments across stages of differentiation. (B) Enrichments across general unsupervised clusters (resolution = 0.05). (C) Enrichments across general ad hoc assignments. (D) Enrichments across osteogenic ad hoc assignments. (E) Enrichments across osteogenic unsupervised clusters (resolution = 0.50). (TIF) [file pgen.1010073.s044.tif]

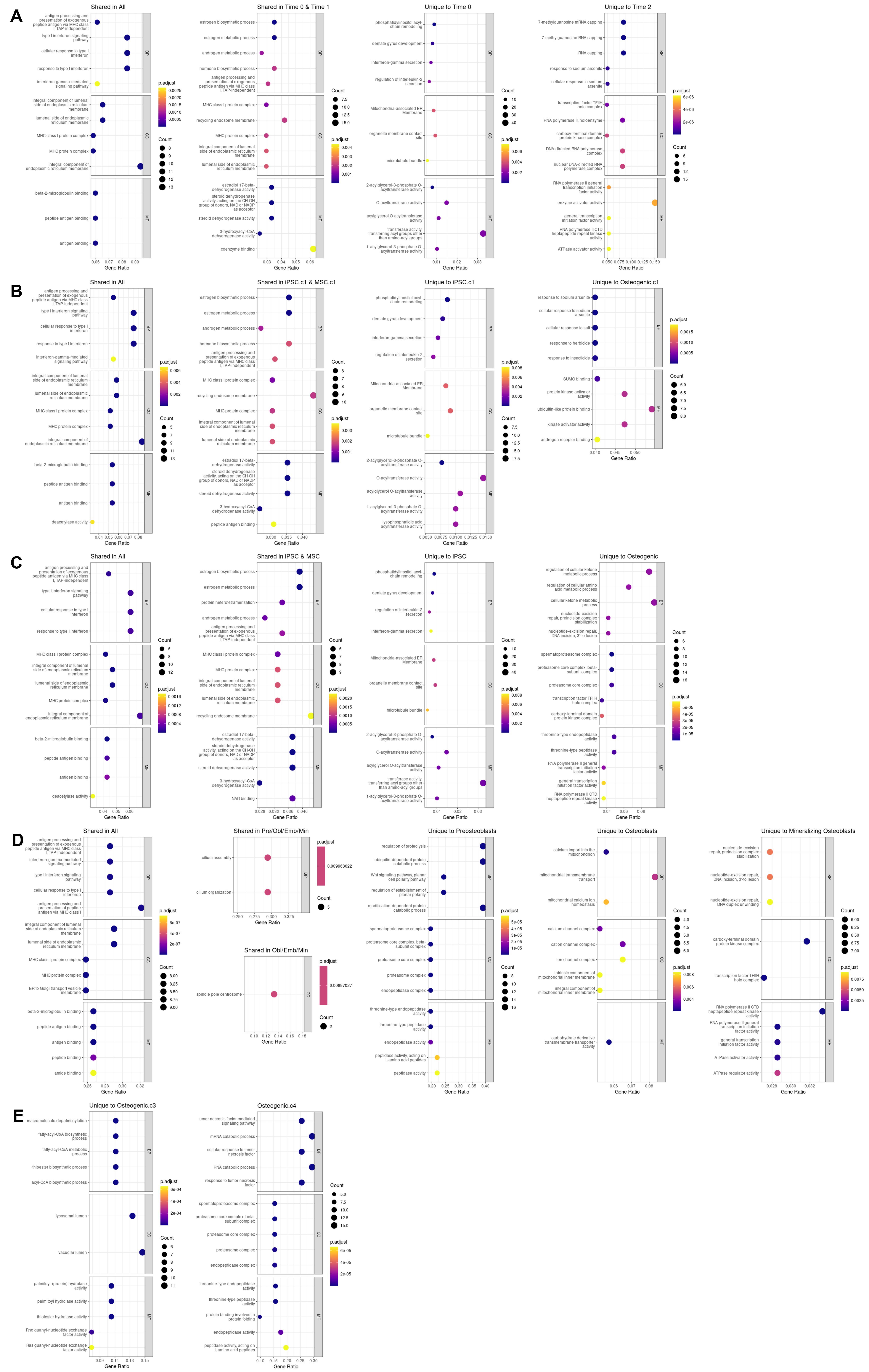

Supplement: S26 Fig — Enrichment of GO functional categories among standard interspecific DE genes (FDR<0.01) identified for a given cell classification. The top 5 GO functions identified in biological processes (BP), cell components (CC), and molecular functions (MF) are displayed along with the adjusted p-value (p-adjust), the number of marker genes overlapping a GO function (Count), and the ratio of overlapping to non-overlapping marker genes for a given GO function (Gene Ratio). (A) Enrichments across stages of differentiation. (B) Enrichments across general unsupervised clusters (resolution = 0.05). (C) Enrichments across general ad hoc assignments. (D) Enrichments across osteogenic ad hoc assignments. (E) Enrichments across osteogenic unsupervised clusters (resolution = 0.50). (TIF) [file pgen.1010073.s045.tif]

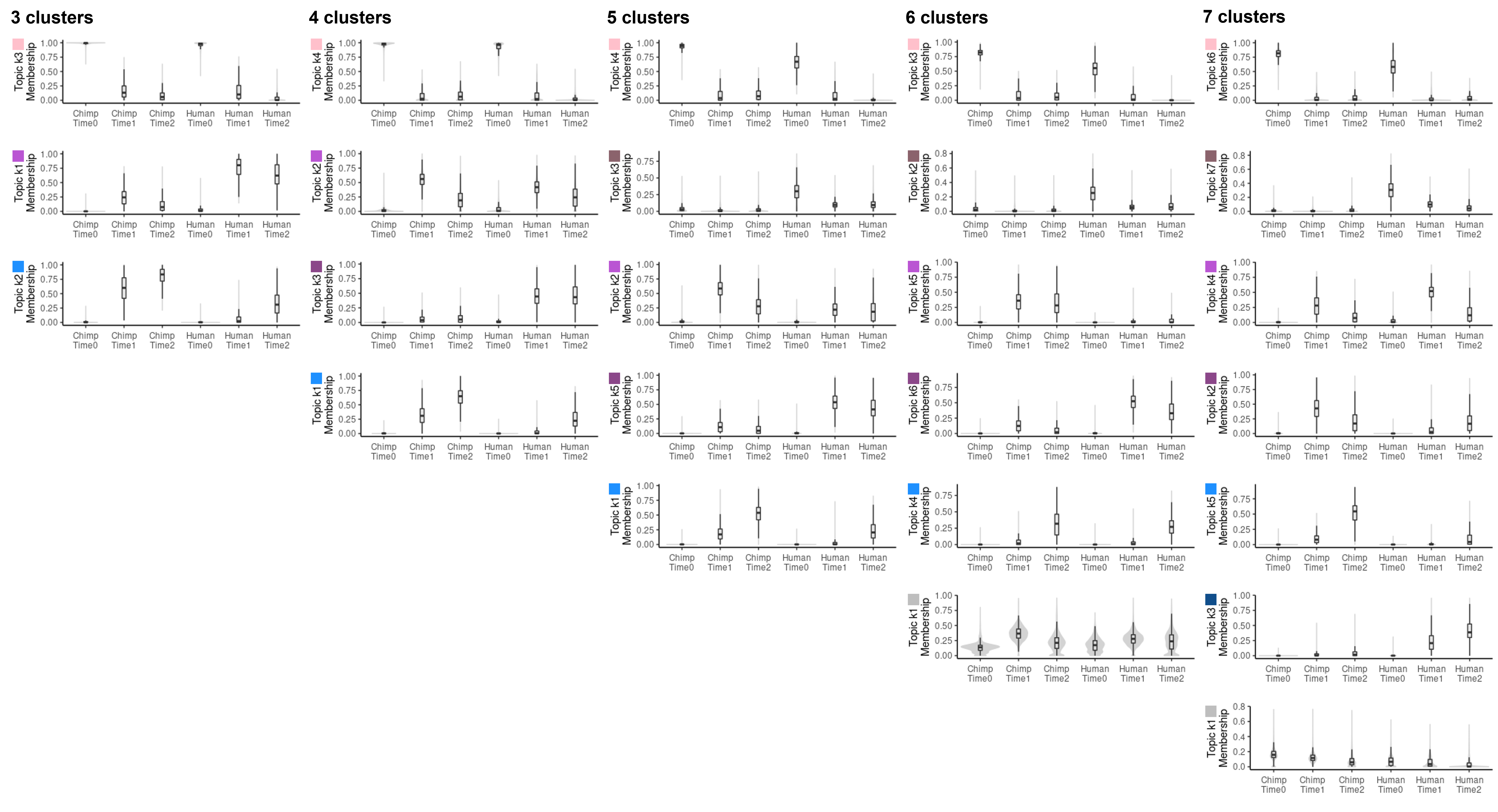

Supplement: S27 Fig — Boxplots showing the grade of membership results of topic modeling at k = 3, k = 4, k = 5, k = 6, and k = 7. Each topic is plotted separately with the grade of membership of cells in a topic noted along the y-axis. Cells are grouped by their species of origin and collection time point. The color denoted by each k references the color of that topic plotted in Fig 5. (TIF) [file pgen.1010073.s046.tif]

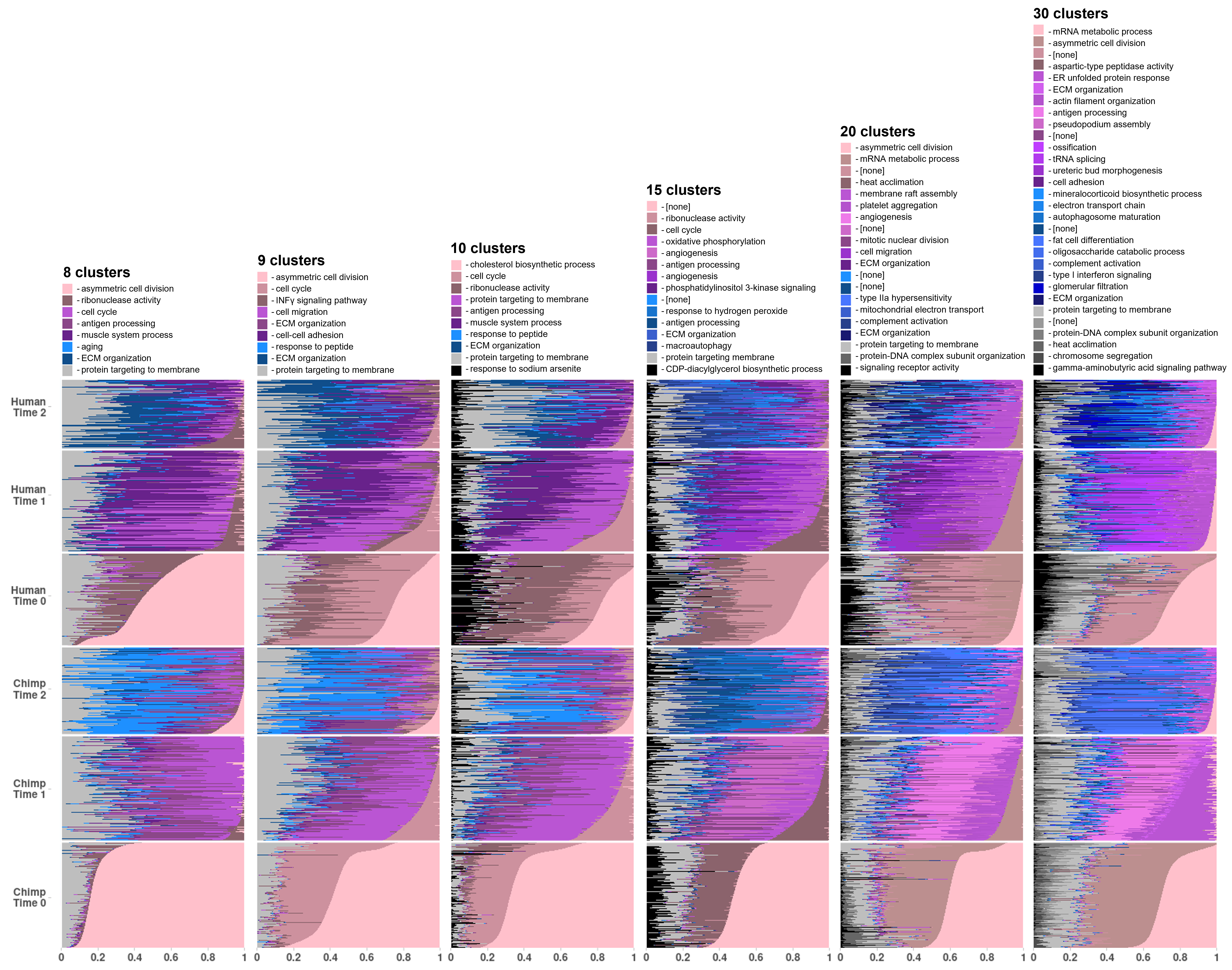

Supplement: S28 Fig — Structure plots showing the results of topic modeling at k = 8, k = 9, k = 10, k = 15, k = 20, and k = 30 with each row representing the gene expression profile from one cell, each colored bar representing a topic, and the grade of membership in each topic depicted by the length of the bar along the x-axis. Cells are grouped by their species of origin and collection time point, and the key notes the top GO category enrichment of marker genes for a given topic. (TIF) [file pgen.1010073.s047.tif]
